# Multi-facility, high-temporal-resolution phenomics of growth–defence allocation responses in potato

**DOI:** 10.1016/j.plaphe.2026.100297

**Published:** 2026-10-22

**Authors:** Sylvain Poque, Murilo A. Sandroni, Pedro Garcia-Caparros, Jesper Cairo Westergaard, Katriina Mouhu, Manzur E. Mohsina Ferdous, Erik Andreasson, Laura J. Grenville-Briggs, Åsa Lankinen, Thomas Roitsch, Kristiina I.H. Himanen, Erik Alexandersson

**Affiliations:** a Department of Agricultural Sciences, University of Helsinki, Finland; b Swedish University of Agricultural Sciences, Department of Plant Protection Biology, Lomma, Sweden; c University of Copenhagen, Department of Plant and Environmental Science, Taastrup, Denmark; d CzechGlobe-Global Change Research Institute CAS, 603 00, Brno, Czech Republic; e Aarhus University, Department of Environmental Science, Roskilde, Denmark; f National Plant Phenotyping Infrastructure, Helsinki Institute of Life Sciences, Biocenter Finland, Department of Organismal and Evolutionary Biology, University of Helsinki, Finland

**Keywords:** Induced resistance, High-throughput physiological phenotyping, Phenomics facility comparison, *Solanum tuberosum*, Growth-defence allocation

## Abstract

Understanding how plants allocate resources between growth and defence is a fundamental question in plant biology. Such allocation responses are often subtle, requiring precise measurement tools and comparative experimental designs to detect. In this study, a high-throughput phenomics framework with daily monitoring was deployed at two independent facilities to assess growth, morphology, and physiology of *Solanum tuberosum* cv. Désirée and six transgenic lines under pathogen-free conditions. Plants treated with resistance-inducing compounds β-aminobutyric acid (BABA) or potassium phosphite (KPhi), alongside lines carrying single *Rpi* genes (R2A, blb1), a three-gene stack (3R1, 3R2), or RNAi knockdown of the jasmonate co-receptor *StCOI1* (coi1H1, coi1X5), were phenotyped to compare the allocation responses of resistance inducers and genetically encoded resistance. Over four weeks, image-derived traits revealed consistent effects across both facilities: BABA increased plant height and elevated the greenness index, whereas KPhi imposed no consistent change in growth; *StCOI1*-silenced lines gained in height. Endpoint enzyme profiling revealed that BABA and KPhi engaged distinct enzyme classes within each facility, with KPhi consistently imposing a lower apparent growth cost; *StCOI1*-silenced lines showed facility-dependent enzyme configurations despite a consistent height phenotype; however, the specific enzyme signatures were not conserved across facilities, indicating that cross-facility enzyme phenotyping requires explicit characterisation of the local metabolic baseline. Overall, high-throughput phenomics resolved subtle, resistance-associated allocation responses across platforms, showing that BABA and KPhi were associated with distinct endpoint enzyme signatures in a given environment, with KPhi's lower cost conserved across environments but signature identity shaped jointly by treatment and environment.

## 1 Introduction

Understanding the allocation trade-offs between plant growth and defence responses is of fundamental importance for both plant biology and crop improvement. Plants can employ different defence strategies against pests and pathogens, ranging from metabolically costly constitutive defences to more energy-efficient triggered defences [1], [2]. For example, cell surface-localized pattern-recognition receptors (PRRs) and intracellular nucleotide-binding leucine-rich repeat receptors (NLRs; also termed R genes) activate PAMP-triggered immunity (PTI) and effector-triggered immunity (ETI), respectively [3], [4]. PTI and ETI are interdependent, activating common signalling components and reinforcing each other to strengthen the overall plant immune response [5], [6], and can both trigger systemic responses in the plant, known as systemic acquired resistance (SAR) [7]. SAR is generally salicylic acid-dependent, while induced systemic resistance (ISR) is microbe-induced and primarily regulated by jasmonic acid and ethylene following root colonisation by beneficial soil microorganisms [7].

Studies of costs of resistance to pathogens over the past 50 years have revealed that trade-offs between defence and growth are often caused by pleiotropic gene effects and antagonistic cross-talk among plant hormones [2], [8], [9]. A study in tomato and potato found little evidence for broad infection-induced transcription of R genes; most R genes were expressed at low levels and only about 10% were differentially expressed during infection [10]. The observation that most R genes are not strongly transcriptionally induced during infection suggests that extensive de novo transcription of R genes is not a major component of the defence response. Consequently, any fitness costs associated with resistance are more likely to arise from constitutive receptor maintenance and/or the downstream defence responses triggered after pathogen recognition. Understanding which downstream processes drive these costs, and whether they are mechanism-specific or shared, is directly relevant for crop improvement strategies such as R gene pyramiding and the use of susceptibility (S) genes [11], [12]. Whether combining multiple R genes amplifies allocation costs by broadening the scope of constitutive immune activation is an open question with direct relevance for pyramiding strategies.

In addition to microbe-triggered ISR, plants can respond to exogenous compounds referred to as resistance inducers (RIs), of which β-aminobutyric acid (BABA) is the most extensively studied [13], [14]. Potassium phosphite (KPhi) has also emerged as a chemical resistance inducer in crop protection [15]. In potato, both compounds can control late blight caused by the oomycete pathogen *Phytophthora infestans* and other diseases [16], [17]. BABA reduces late blight lesions by up to 50% in greenhouse-grown plants [16], while KPhi provides field protection matching full fungicide applications in resistant varieties [18], [19]. Both compounds activate defence-related genes, resulting in an IR phenotype [20], [21], and alter the abundance of metabolites, transcripts, and proteins [22], [23]. However, fitness costs associated with IR can be difficult to quantify without large sample sizes or accurate measurements under constant environmental conditions [24]. Studies in *Arabidopsis* have shown that BABA induction, even without pathogen challenge, can reduce growth and seed production, particularly at higher doses [25]. Such costs may be more pronounced in model plants than in crops, since crop breeding may have selected alleles that confer defence benefits at lower cost [8], [26]. Assessing allocation responses associated with IR directly in crops is therefore important to determine whether IR can be deployed for protection without compromising crop yield.

An underexplored question is whether different defence mechanisms, chemical inducers versus genetically encoded resistance, engage the same or distinct metabolic reallocation routes. Such reallocation can arise from competition for resources between growth and defence and from the activation of direct or indirect defence responses. Source/sink relations and defence mechanisms have been shown to be co-ordinately regulated in response to pathogen-related stimuli [27] and to pathogen infection [28]. Since enzymes of carbohydrate metabolism operate at key branch points of carbon flux, their activity signatures provide a biochemical readout of how resources are partitioned between growth and defence. Cell physiological phenotyping, based on activity signatures of key enzymes of carbohydrate and antioxidant metabolism [29], has proven a valuable approach to characterise the impact of pathogen infection [30], [31] and has also been proposed as a proxy for the cost of defence [32]. Resolving these allocation routes therefore requires systematic growth monitoring combined with cell-physiological resolution of carbon and antioxidant metabolism [33]. It remains unclear, however, whether the same macroscopic growth-defence trade-off, when observed across contrasting environments, is underpinned by the same metabolic reallocation routes, or whether equivalent phenotypic outcomes can arise through distinct biochemical configurations [34], [35]. This question is particularly relevant in multi-environment studies, where local conditions may independently modulate carbon metabolism, potentially generating environment-specific enzyme biosignatures even when macroscopic phenotypes are consistent across sites.

High-throughput plant phenotyping in controlled climates represents an ideal tool to objectively monitor plant performance with high temporal resolution and quantify defence-related costs. Yet, this approach has rarely been applied to quantify the fitness costs of resistance mechanisms [36]. To better characterise the potential fitness costs of resistance in an agriculturally relevant crop, we conducted experiments in two Nordic plant phenotyping facilities: the PhenoLab at the University of Copenhagen and the National Plant Phenotyping Infrastructure (NaPPI) at the University of Helsinki [37]. The cross-facility design was used to assess whether growth-defence trade-offs are detectable at both macroscopic and metabolic resolution, through which routes resources are reallocated, and whether such patterns are consistent across contrasting environments.

In the present study, we combined time-series imaging with chemical resistance inducers and a mechanistically diverse set of transgenic potato lines to directly compare whether resistance inducers and genetic resistance mechanisms impose similar fitness costs and engage the same metabolic reallocation routes under pathogen-free conditions. BABA and KPhi, two chemically distinct resistance inducers with different modes of action, were applied to potato (cv. Désirée) to test whether chemical induction of resistance engages a common or inducer-specific reallocation route. To assess costs associated with R gene expression, four transgenic lines carrying single (*R2A*, *blb1*) or stacked (*3R1*, *3R2*) *Rpi* genes were included [38]. To test whether disruption of jasmonate signalling affects plant fitness, two RNAi lines with reduced *StCOI1* expression were also evaluated. Specifically, image-based sensors were used to track canopy area, plant height, morphological traits, and canopy colour over four weeks of growth. Physiological costs were assessed using photosynthetic parameters, including PSII maximum quantum yield, as a proxy for photosynthetic activity. Endpoint measurements included above-ground biomass, tuber yield, and activity signatures of key enzymes of carbohydrate and antioxidant metabolism. Integration of multi-sensor and biochemical layers allowed us to distinguish mechanism-dependent allocation routes from shared macroscopic trade-off signatures, and to evaluate which phenotypic endpoints are reproducible across contrasting growing environments.

## 2 Materials and methods

### 2.1 Experimental design

The overall experimental design is summarised in the graphical abstract (Fig. 1). Time-series phenotyping data were analysed using generalized additive mixed models (GAMMs); enzymatic profiles were analysed by principal component analysis (PCA).

**Fig. 1:**
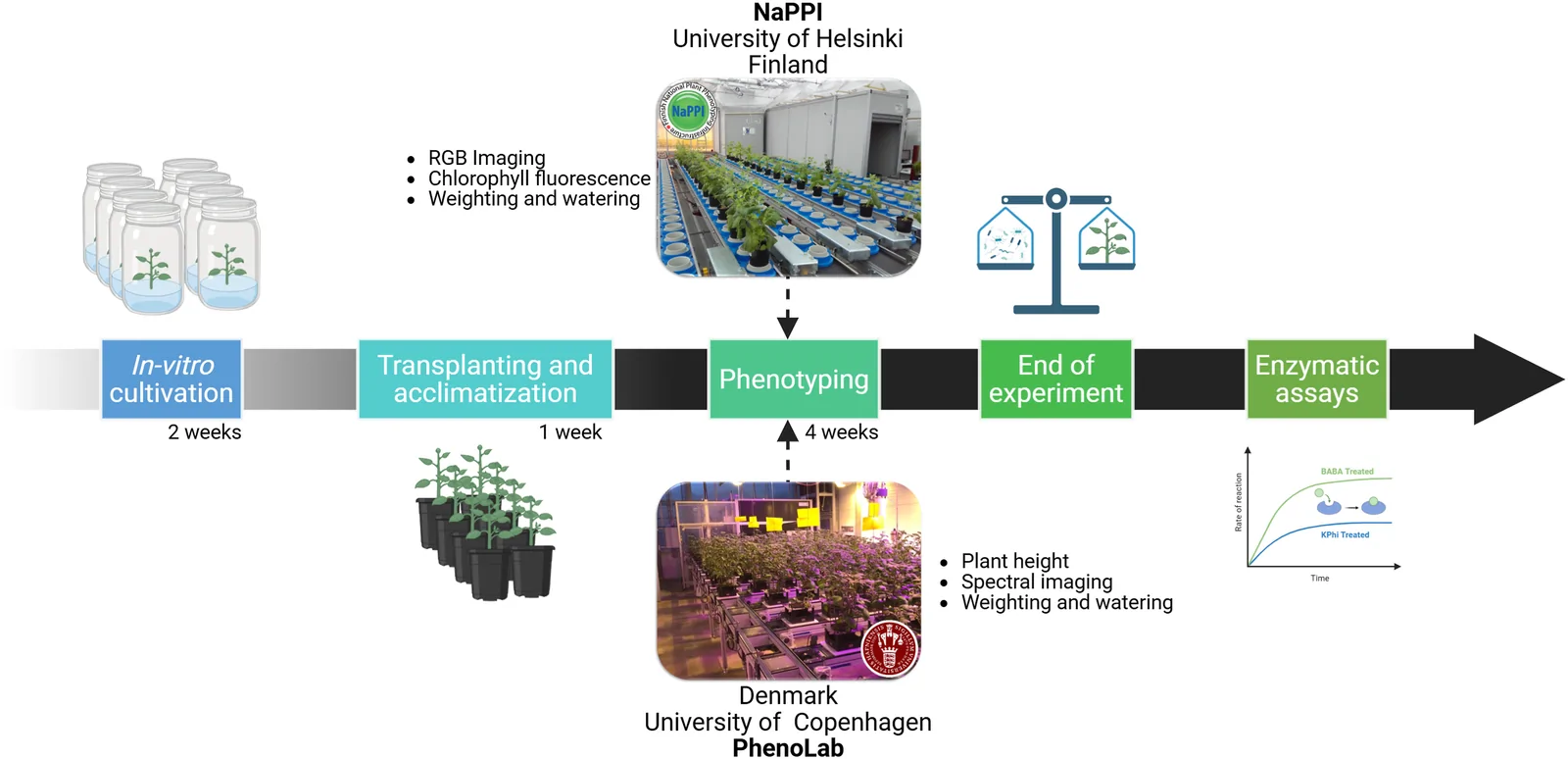
Experimental workflow at the high-throughput plant phenotyping platforms. Created in BioRender. Poque, S. (2026) https://BioRender.com/nmkve7g.

### 2.2 Plant material

*Solanum tuberosum* cv. Désirée was used as a common reference in all experiments. It was selected for its moderate susceptibility to *P. infestans*. Six transgenic potato lines, all generated in the Désirée background (Table 1), were also evaluated: two independent overexpression lines for single *Rpi* genes, namely R2A (overexpressing the race-specific *R2* gene [39]) and blb1 (overexpressing the broad-spectrum late-blight resistance gene *Rpi-blb1* [40]); two lines (3R1 and 3R2) carrying a stack of three *Rpi* genes, *Rpi-blb1*, *Rpi-blb2* and *Rpi-vnt1.1* [38], [41]; and two independent RNAi knockdown lines for the jasmonate co-receptor *StCOI1*, coi1H1 and coi1X5, with reduced sensitivity to jasmonic acid methyl ester [42]. Both R2 and Rpi-blb1 encode intracellular nucleotide-binding leucine-rich repeat (NLR) immune receptors that recognise a corresponding *P. infestans* effector protein and trigger effector-triggered immunity (ETI). R2 recognises a race-specific effector of the Avr2 family. Rpi-blb1, originally identified in the wild species *Solanum bulbocastanum*, recognises the broadly conserved IPI-O/Avrblb1 effector and, consequently, tends to give a wider resistance spectrum than R2. In the 3R1 and 3R2 lines, each of the three stacked genes recognises a distinct *P. infestans* effector (Avrblb1, Avrblb2, and Avr-vnt1, respectively). Combining three independently acting NLR receptors in a single line is a pyramiding strategy intended to broaden the effective resistance spectrum and to reduce the likelihood of resistance being overcome by effector loss or diversification in the pathogen population. StCOI1 encodes the F-box component of the SCF^COI1^ ubiquitin ligase, which perceives bioactive jasmonate. Upon activation, this complex targets JAZ repressor proteins for degradation, thereby de-repressing jasmonate-responsive defence genes. The same pathway is also implicated in the growth-repressive side of jasmonate signalling. Silencing StCOI1 was therefore used here to test whether disrupting the defence-signalling hub itself, rather than activating a specific resistance gene, carries a detectable growth-defence allocation cost. Six biological replicates were used per line and per resistance-inducer treatment. At NaPPI, this gives 54 plants: 36 from the six transgenic lines under Control only (six lines × six replicates), plus 18 from Désirée under Control, BABA, and phosphite treatment (three treatments × six replicates); the six Désirée/Control plants serve as the shared reference for both the genotype and the induced-resistance comparisons. The PhenoLab experiment followed the same design, also giving 54 plants.

**Table 1:** Potato cultivar and transgenic material grown at the National Plant Phenotyping Infrastructure (NaPPI) and the PhenoLab with their known late blight resistance and original references.

Genotype	Late blight resistance	Reference
***Cultivar***		
**Désirée**	Moderately susceptible	[43]
***Transgenic lines in Désirée (cv.)***		
**3R1**	Expression of Rpi-blb1 and Rpi-blb2 from *Solanum bulbocastanum*	[41]
	Rpi-vnt1.1 from *Solanum venturii*	
**3R2**	Expression of Rpi-blb1 and Rpi-blb2 from *Solanum bulbocastanum*	[41]
	Rpi-vnt1.1 from *Solanum venturii*	
**coi1H1**	Reduced sensitivity to jasmonic acid methyl ester	[42]
**coi1X5**	Reduced sensitivity to jasmonic acid methyl ester	[42]
**R2A**	Expression of a R2 resistance gene Rpi-ABPT	[39]
**blb1**	Expression of the broad-spectrum resistance gene Rpi-blb1	[40]

### 2.3 Plant growth conditions

The experiments were conducted across two high-throughput plant phenotyping (HTPP) facilities to increase analytical capacity and to complement the analysis with the different sensors available at each location. The National Plant Phenotyping Infrastructure (NaPPI) at the University of Helsinki, Finland, is housed in the Viikki Plant Growth Facilities, and the second HTPP facility, the PhenoLab is part of the University of Copenhagen, Denmark.

The first experiment was conducted during spring 2019 in the NaPPI. *In-vitro* plantlets were generated from cuttings at SLU, Alnarp, Sweden. Plants were grown under sterile conditions for two weeks in tissue culture in a Phyto chamber with a day length of 16 h (light conditions: 140 μmol m^−2^ s^−1^) at a temperature of 22 °C. The plantlets were then transported to NaPPI in their *in-vitro* containers and, upon arrival, were transplanted firstly to small pots of 8 cm diameter for *ex-vitro* acclimation. After a week, the seedlings were transferred to 5 L plastic pots filled with “Kekkilä Karkea Ruukutusseos W R8014” (Kekkilä, Finland) and brought to the automated conveyor and imaging platform of the facility (see *RGB image acquisition and trait extraction* below) for four weeks. Automated watering was controlled using pot weighing, allowing daily recording of pot weights and maintaining each plant at a target total weight of 3.3 kg. The plants were fertigated once with 2 μS Kekkilä Vegetable-Suberex solution (Kekkilä, Finland) at the beginning of the experiment.

The environment was programmed to an 18-h photoperiod with target temperatures of 20 °C during the day and 18 °C at night. Environmental parameters were recorded at 1-min intervals and included air temperature, relative humidity (RH), and light intensity (LI). Based on the recorded data, the actual mean daytime temperature was 25.6 °C, with a maximum of 35.9 °C and a minimum of 18.7 °C. During the night, the mean temperature was 21.1 °C, with maximum and minimum values of 30.1 °C and 18.6 °C, respectively. Mean daytime RH was 37.5%, while nighttime averaged 48.8%. When natural light intensity fell below the target threshold, supplemental illumination was supplied using high-pressure sodium (HPS) lamps, resulting in a mean daytime light intensity of 248.8 μmol m^−2^ s^−1^. Daytime light intensity ranged from 11.0 to 1868.0 μmol m^−2^ s^−1^.

For the second experiment, in the PhenoLab HTPP facility autumn 2019, the *in-vitro* plantlets were transplanted into 1.5 L plastic pots filled with a commercial pot soil (Yrkesplantjord, Weibulls, Sweden) and grown in a controlled environment chamber at Biotron, SLU Alnarp, at 20 °C, 65% RH, and a photoperiod of 16 h of light (160 μmol m^−2^ s^−1^) for one week before they were transferred to the fully automated plant phenotyping facility PhenoLab at the University of Copenhagen Taastrup campus [44]. The seedlings were transplanted to square pots, 13 × 13 cm, filled with 2.2 L of commercial substrate (Krukväxtjord Lera & Kisel, Hammenhög, Sweden) supplemented with PG-Mix powder fertilizer. The seedlings were grown under natural greenhouse light supplemented with white tubular Osram LED lamps to provide a 16-h photoperiod, with light intensity (at 150 mm) of 120 μmol m^−2^ s^−1^, day/night temperatures of 20/18 °C, and 70% relative humidity. Automated watering and imaging sessions in the morning and afternoon, as well as randomization, were carried out every 6 h for four weeks. Environmental parameters were recorded at 3-min intervals and included air temperature, relative humidity, and light intensity. Based on the recorded data, the actual mean daytime temperature was 22.8 °C, with a maximum of 25.1 °C and a minimum of 17.9 °C. During the night, the mean temperature was 18.8 °C, with maximum and minimum values of 24.3 °C and 17.4 °C, respectively. Mean daytime RH was 56.9%, while nighttime averaged 57.3%. Illumination was provided using LED lamps, resulting in a mean daytime light intensity of 224.3 μmol m^−2^ s^−1^. Daytime light intensity ranged from 27.4 to 472.3 μmol m^−2^ s^−1^.

### 2.4 Induced resistance (IR) treatments

For resistance-inducer (RI) treatments, Désirée plants were used and the same Désirée plants served as control for both the line set and the RI treatment set. The IR treatments started seven days after plantlets were transferred to the two phenotyping facilities and were carried out on a weekly basis. Plants were sprayed once a week on four occasions, with 10 mL of tap water (control), 10 mL of BABA 10 mM (DL-3-aminobutyric acid, Sigma A44207) or 10 mL of 1.25% (v/v) Proalexin (LMI AB, Helsingborg, Sweden), corresponding to 36 mM of potassium phosphite (KPhi).

### 2.5 RGB image acquisition and trait extraction

At NaPPI, automated imaging was carried out on the Modular Plantscreen™ Conveyor System (Photon Systems Instruments, Drásov, Czech Republic), with a capacity of 270 plants. For the first experiment conducted in NaPPI, visible light Red Green Blue (RGB) top (RGB2) and side view (RGB1) imaging was performed every second day for top view and once a week for side view during the experiment to collect data on growth in area and height, morphological traits, and colour. Side- and top-down view images were captured using an RGB line scan and top-down cameras (IDS Imaging Development Systems GmbH, Obersulm, Germany), respectively. Top view RGB images of the plants were captured at resolution 2560 × 1920 pixels and camera height was automatically adjusted by a light-curtain unit measuring plant height. For side view images, the line scan camera captured images at a resolution of 2560 × 3476 pixels. The RGB images of each individual plant were processed by first removing the background using pixel-based colour thresholding, followed by the extraction of morphological parameters and colour-based segmentation using the MorphoAnalysis v. 1.0.5.1 software (Photon Systems Instruments, Drásov, Czech Republic). During the experiment, plant height was determined using two methods: weekly extraction from the side-view images (RGB1) and daily measurements from a light curtain. For the RGB1 measurement, three side-view images were taken at each time point, with a 120° rotation of the plant between each imaging. Plant height was then calculated as the average of values obtained from the three angles. Plant canopy area and greenness were quantified from top-view images (RGB2), with the GREENNESS parameter computed using Python version 3.10.7 and OpenCV [45]. For each image, the pixel values of the Red (R), Green (G), and Blue (B) channels were averaged to obtain mean values (avgR, avgG, avgB). The GREENNESS parameter was then calculated using the formula described by Lazarević et al. [46]:

(1) $$GREENNESS=\frac{2\times avgG-avgR-avgB}{2\times avgR+avgG+avgB}$$

### 2.6 Photosystem II photochemistry measurements

Chlorophyll fluorescence (ChlF) measurements were carried out in the NaPPI facility using a top-view Pulse Amplitude Modulated (PAM) chlorophyll *a* fluorometer (FluorCam FC-800MF, PSI, Czech Republic) equipped with a dark-adaptation tunnel [47]. ChlF imaging was used to monitor plant physiological responses and detect stress indicators at the end of the experiment. ChlF parameters were measured after a 15-min dark-adaptation period using a quenching protocol developed in FluorCam 7.0 software (PSI, Drásov, Czech Republic). The quenching protocol comprised two phases: a dark-adapted phase followed by a light-adapted (actinic) phase. In the dark-adapted phase, minimal fluorescence (F_0_) was recorded and a saturating pulse was applied to obtain maximal fluorescence (F_m_); variable fluorescence was determined as F_v_ = F_m_-F_0_, and the maximum PSII quantum yield as F_v_/F_m_. In the subsequent light-adapted phase, actinic light was applied, allowing measurement of F_p_, the fluorescence level immediately after light onset, while saturating pulses were used to determine maximal fluorescence in the light (F_m__Lss) and the steady-state fluorescence (F_t__Lss). The effective PSII quantum yield at light steady state was calculated as QY_Lss=(F_m__Lss-F_t__Lss)/F_m__Lss. To evaluate non-photochemical dissipation under actinic light, steady state nonphotochemical quenching was computed as NPQ_Lss=(F_m_-F_m__Lss)/F_m__Lss and the ratio of fluorescence decline at light steady state as Rfd_Lss=(F_p_-F_t__Lss)/F_t__Lss. A higher Rfd_Lss indicates a stronger decline from initial to steady-state fluorescence, reflecting greater activation of dissipative processes; importantly, the related Rfd is widely used as a vitality index as it correlates with photosynthetic performance and overall plant condition [48], [49].

### 2.7 Multispectral imaging

For the second experiment conducted in PhenoLab, imaging was performed using a multispectral system capturing ten discrete spectral bands from UVA to NIR. All images were acquired from the top view only. The system used a JAI BM 500 GE camera equipped with a Sony ICX625 2/3″ CCD monochrome sensor (2456 × 2058 pixels). Plants were illuminated sequentially in ten narrow spectral bands, each 10–20 nm wide, covering the range of 365–970 nm. This setup enabled the acquisition of calibrated reflectance data across ten image layers for each plant as it passed through the imaging station.

The acquisition mode alternated between crop coverage (CC) priority and reflectance priority, with the priority mode typically switched at midday on weekdays. Imaging was scheduled twice daily for four weeks, and the plants were randomized in the greenhouse after each imaging session. Throughout the trial, quality control images were collected to detect overexposure and other imaging artefacts that required correction.

Post-trial, all multispectral images were processed in VideometerLab version 3.12.25 (Videometer, Herlev, Denmark) to generate segmentation algorithms isolating plant tissue [50]. These segmentations were used to extract crop coverage and band-specific reflectance values. NDVI was calculated from pixel-wise reflectance values, with avgNIR computed as the average reflectance of the three NIR channels (780, 890, and 970 nm) and avgRED as the average reflectance of the red channels (645 and 670 nm):

(2) $$NDVI=\frac{avgNIR-avgRED}{avgNIR+avgRED}$$

To enable comparison with NaPPI-derived data, plant greenness in PhenoLab was computed using the same GREENNESS metric described above, where avgG corresponds to the green 525 nm band, avgB to the blue 460 nm band, and avgR to the average of the two red bands at 645 and 670 nm.

Due to the physical constraints of the PhenoLab system, approximately 60 cm in height clearance and 40 cm in width, the crop coverage (CC) imaging, which is always performed with the camera positioned 60 cm above the pot surface, is only relevant for part of the growth period. As plants grew, most exceeded the system's imaging-area limitation, so a reliable crop-coverage reading was only available during the early part of the growth period. Since the allocation responses of interest were expected to develop over the full growth period rather than only at its outset, this restricted window was unlikely to capture them; thus, the trait was not reported in this study. Plant height was measured twice daily using a line scan light curtain with 2D snapshot technology (Model VSPM-6F2413, AG, Waldkirch, Germany) positioned before entry into the vision chamber.

### 2.8 Biomass measurements at the end of the experiment

At harvest (approximately 38 days after transplanting, DAT) at both facilities, plants were weighed for above-ground fresh matter (FM, in g), and tubers were counted (number of tubers, NT) and weighed (tuber weight, TubW, in g). Plant shoots were bagged and dried at 75 °C for three days, after which above-ground dry matter (DM, in g) was recorded. All six biological replicates per group were assessed at both facilities, except the NaPPI Désirée/Control group, where two plants could not be accurately weighed due to a handling issue during harvest; above-ground fresh and dry weight were therefore recorded for four of the six plants in this group, while tuber counts and weights were unaffected and recorded for all six.

### 2.9 Enzymatic assays

At the end of the two experiments, four to six leaflets were sampled from the top of the plant (third to sixth leaf) and quickly frozen in liquid nitrogen. Samples were kept in −80 °C until further processing. For the enzymatic assays, frozen potato leaves were homogenized in liquid nitrogen and extraction was done according to the protocol reported by Ref. [51]. A total of 500 mg of ground plant material from each sample was mixed with 1 mL of extraction buffer (40 mM TRIS-HCl pH 7.6, 3 mM MgCl_2_, 1 mM EDTA, 0.1 mM PMSF, 1 mM benzamidine, 14 mM β-mercaptoethanol, 24 μM NADP) for 60 min at 4 °C. The homogenate was centrifuged at 4 °C and 20,000 *g* for 45 min. The supernatant (crude extract) was aliquoted to new tubes and kept on ice. The pellet was washed three times with distilled water, re-suspended in 1 mL of high salt buffer (1 M NaCl, 40 mM TRIS-HCl pH 7.6, 3 mM MgCl_2_, 1 mM EDTA, 0.1 mM PMSF, 1 mM benzamidine, 14 mM β-mercaptoethanol, 24 μM NADP), and mixed by continuous shaking at 4 °C overnight. The homogenate was centrifuged at 4 °C and 20,000 *g* for 25 min and the supernatant (cell wall extract) was transferred to new tubes. Both the cell wall extract and the crude extract were dialysed overnight with 20 mM potassium phosphate buffer (pH 7.4) at 4 °C. The extracts were aliquoted, frozen in liquid nitrogen, and stored at −20 °C until further use.

For the estimation of carbohydrate enzyme activities, the protocol described by Jammer et al. [51] was followed, while antioxidant enzyme activities were measured following the method of Fimognari et al. [52]. Carbohydrate-metabolising enzymes were assayed because they act at key entry, branch, and exit points of central carbon metabolism and thereby report how carbon is partitioned between growth-related sinks (e.g., starch and cell-wall biosynthesis) and defence-related metabolic demand, consistent with the cell-physiological phenotyping framework used to characterise growth-defence trade-offs in other pathosystems [29], [30], [31], [32]. Antioxidant enzymes were assayed because reactive oxygen species (ROS) production and detoxification are a core, energetically costly component of both pattern- and effector-triggered plant immune responses, providing a complementary proxy for the defence-signalling arm of the trade-off. A 96-well microtiter plate (Sarstedt, Nümbrecht, Germany) was used for the semi-high-throughput analysis of kinetic assay of C enzymes: phosphoglucose isomerase (PGI, EC 5.3.1.9), phosphoglucomutase (PGM, EC 2.7.5.1), UDP-glucose pyrophosphorylase (UGPase, EC 2.7.7.9), ADP-glucose pyrophosphorylase (AGPase, EC 2.7.7.27), hexokinase (HKX, EC 2.7.1.1), fructokinase (FK, EC 2.7.1.4), phosphofructokinase (PFK, EC 2.7.1.11), sucrose synthase (Susy, EC 2.4.1.13), aldolase (Ald, EC 4.1.2.13), cell wall invertases (cwInv, EC 3.2.1.26), vacuolar invertase (vacInv, EC 3.2.1.26) and cytosolic invertase (cytInv, EC 3.2.1.26), and A enzymes: glutathione reductase (GR, EC 1.8.1.7), monodehydroascorbate reductase (MDHR, EC 1.6.5.4), catalase (CAT, EC 1.11.1.6), dehydroascorbate reductase (DHAR, EC 1.8.5.1), glutathione S-transferase (GST, EC 2.5.1.18), guaiacol peroxidase (POX, EC 1.11.1.7), ascorbate peroxidase (APX, EC 1.11.1.11), and apoplastic class III peroxidases (aPOX, EC 1.11.1.7), with 2–3 μL protein extract. For each sample, determinations were done with three technical replicates and mean values are shown for the biological replicates. Three biological replicates with three technical replicates each were analysed per treatment or line at each facility (except for Désirée at PhenoLab, for which only two biological replicates were analysed). Across biological replicates, the coefficient of variation (CV) had a median of 10.9% (mean 14.1%, range 0.5-57.5%) over all 20 enzymes and all groups.

### 2.10 Data processing for detecting outliers

Time series phenotypic data were cleaned using the statgenHTP [53] package in R (version 4.3.3, https://www.R-project.org/). Outlier detection followed the package's built-in workflow, which evaluates the consistency of each observation relative to neighbouring observations within the plant-level trajectory. The procedure applies local regression to approximate each time series using parametric functions. A local regression model is first fitted at selected anchor points along the time course and then interpolated across intermediate points to obtain a smooth expected trajectory. Confidence intervals around the fitted values are then computed, and any observations falling outside these intervals are flagged as outliers. Data points identified as outliers were subsequently removed prior to all downstream analyses. This procedure was applied to plant height and canopy area; no outlier removal was applied to the vegetation index (Greenness, NDVI) data at either facility, and no observations were removed from this dataset. Endpoint enzyme-assay, chlorophyll fluorescence, and tuber/shoot biomass measurements are single readings per plant rather than a time series, so this statgenHTP-based approach does not apply to any of them. For the enzyme data, outlying replicate values were instead identified by visual assessment and excluded, affecting 6 of 1020 enzyme-by-replicate values (0.6%), confined to three invertase-family enzymes and reducing the transgenic-line PCA sample size accordingly; no outlier removal was applied to chlorophyll fluorescence or tuber/shoot biomass. Per-trait detection parameters, exact exclusion counts, and reasons for exclusion are given in Supplementary Table S2: plant height exclusions used confIntSize = 3 (NaPPI, 52 points) or confIntSize = 5 combined with a whole-series slope-consistency criterion (PhenoLab, 358 points, including one entire plant); canopy area exclusions used confIntSize = 3 (NaPPI, 70 points).

### 2.11 Principal component analysis

PCA were performed using Python (version 3.13, http://www.python.org) on standardized enzyme measurements. Prior to analysis, rows containing missing values were removed to ensure a complete numeric matrix. The transgenic-line PCA sample size was reduced due to removal of outlying replicate values in the enzyme-assay data. All enzyme variables were z-scored using the StandardScaler transformer from scikit-learn [54]. A two-component PCA model was then fitted to the scaled data. Sample scores for the first two principal components (PC1 and PC2) were used to visualize treatment-dependent separation among samples, while loading vectors were overlaid to show the contribution and direction of each enzyme in the biplot. All data handling and preprocessing were performed using pandas [55], and PCA biplot visualizations were generated using matplotlib [56] and seaborn [57]. Each treatment or genotype group is represented by three biological replicates (two for Désirée at PhenoLab), each the mean of three technical replicate measurements. In addition, each enzyme was tested individually by one-way ANOVA within each of the four comparisons (induced-resistance treatments and transgenic lines, at each facility), with Benjamini-Hochberg correction across the 20 enzymes of each comparison.

### 2.12 Statistical analysis

Generalized additive mixed models (GAMMs) were used to analyse time-series data, capturing non-linear temporal trends and group-specific trajectories. Time (days after transplanting, DAT) was modelled with penalized splines, with smooth terms varying by IR treatment or transgenic line to allow distinct temporal responses across groups. To account for the experimental design, we included time-varying individual-plant effects using factor-smooth interactions. In the NaPPI facility, block (Block 1: 25 plants; Block 2: 23 plants, including six plants of a further cultivar, Bintje, grown in the same experiment and not reported here) was included as a factor-smooth interaction term to account for spatial growth differences arising from plant placement within the greenhouse. Because residuals in repeated-measures data are typically autocorrelated, we incorporated an AR(1) correlation structure, whose parameter was set to the lag-1 residual correlation within plants of an initial fit without it, and fitted all models using REML with automatic smoothness selection in the R package mgcv [58], through its bam function. Model adequacy was evaluated using standard GAM diagnostics and inspection of residual autocorrelation. The basis-dimension check (k.check) flagged 9 of the 12 GAMMs reported in this manuscript as potentially under-flexible; refitting these 9 models with substantially increased basis dimension changed fitted values by 0.5-4.2% of each trait's data range, with fitted-value correlations relative to the original fit of 0.998-1.000, indicating the reported temporal trends and significant-difference windows are not materially sensitive to this choice. Fitted smooths and pairwise smooth differences, comparing transgenic lines to the Désirée line, or IR treatments to untreated Désirée, were visualized using the R package itsadug [59]. Group differences were evaluated pointwise across the time course from the 95% confidence interval of the difference smooth, without a simultaneous-inference or multiplicity correction across the many timepoints tested. Repeated imaging increases the temporal resolution of each plant's trajectory but not the number of independent biological replicates, which remained six plants per group. To guard against this, all group comparisons underlying the pointwise-CI figures were also evaluated using itsadug's simulation-based simultaneous (family-wise) 95% confidence band, which corrects for testing the difference smooth across the full time course rather than at a single timepoint. The pointwise-significant window, simultaneous-significant window, and effect estimate with 95% confidence interval at the point of maximum divergence are reported for every GAMM comparison in Supplementary Table S4. Single timepoint traits, dry and fresh matter, tuber number and weight, as well as ChlF parameters for transgenic lines from the NaPPI facility were analysed using a linear mixed effects model (R package lme4, [60]) with Genotype as a fixed effect and Block as a random intercept to capture greenhouse placement-related growth differences. Where the mixed model was singular (NaPPI genotype dry weight), a fixed-effects linear model was used instead. For other datasets (e.g., NaPPI IR and all PhenoLab), where a block structure was absent, a fixed effects linear model from base R's stats package was used to estimate genotype or treatment effects for all single-timepoint traits. For all analyses, estimated marginal means (EMMs) followed by Dunnett-type contrasts were used to compare each transgenic line or IR treatment with its designated control, with Bonferroni adjustment for multiple comparisons (R package emmeans, [61]). Endpoint sample sizes were not uniformly six replicates per group for all traits; exact per-group n, means, SDs, and effect estimates are given in Supplementary Table S3. Throughout this manuscript, a nonsignificant result (from either the GAMM pointwise comparisons or the Dunnett-type endpoint contrasts) is reported only as a failure to detect a statistically significant difference at the stated sample size and threshold, not as evidence that no effect exists. All plotting was performed with the R package ggplot2 [62].

## 3 Results

We employed a multi-sensor, multi-line phenomics framework to dissect growth-defence allocation responses in potato, comparing chemical resistance inducers and transgenic lines with distinct immunity mechanisms across two independent high-throughput phenotyping facilities. Plants were assessed for growth, development, and physiological traits over a period of four weeks.

### 3.1 Induced resistance (IR) treatments

We evaluated potential costs associated with applying plant resistance inducers β-aminobutyric acid (BABA) and potassium phosphite (KPhi) to plants grown in two distinct high-throughput phenotyping (HTPP) facilities.

#### 3.1.1 Plant height following IR treatments over the course of the experiment

Plant height was monitored daily by image-based sensors at both NaPPI and PhenoLab throughout the experiment. BABA-treated plants grew taller than control plants at both facilities, based on non-overlapping GAM-derived 95% confidence intervals (Fig. 2), with the height difference reaching +86.3 mm (95% CI 59.8 to 113) at 38 DAT in NaPPI and +131 mm (95% CI 101 to 161) at 34 DAT in PhenoLab; effect estimates and 95% confidence intervals at the point of maximum divergence are given for all GAMM comparisons in Supplementary Table S4. KPhi-treated plants did not differ significantly from the control at either facility. The BABA-associated increase in plant height was thus consistent across both facilities.

**Fig. 2:**
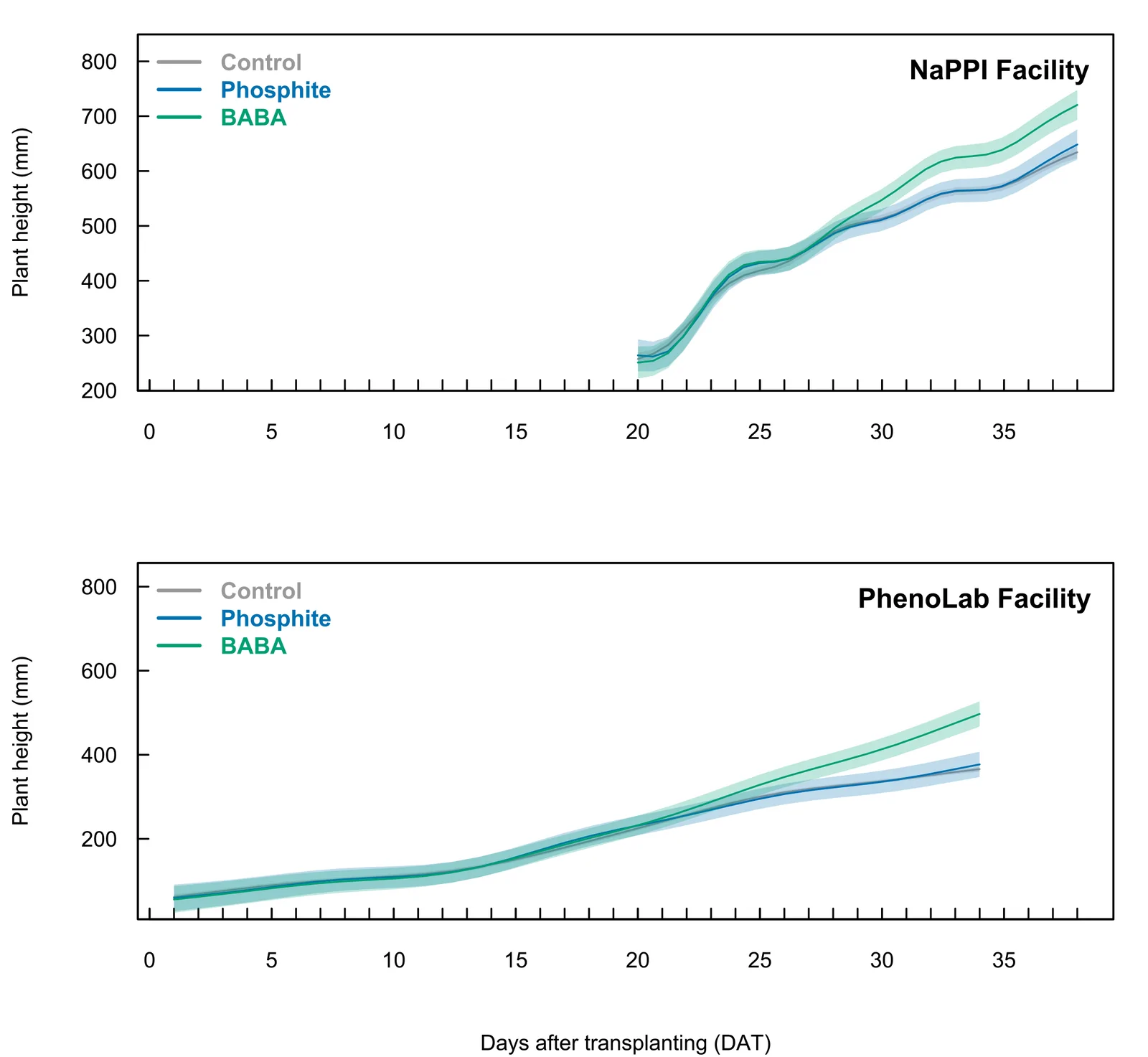
Plant height dynamics in response to induced-resistance treatments. Generalized Additive Mixed Models (GAMMs) were used to analyse plant height measured in NaPPI (upper panel) and PhenoLab (lower panel). Group differences were evaluated using two-sided pairwise smooth-difference tests derived from the GAMMs, with significance concluded when the 95% confidence interval of the difference did not include zero. Six biological replicates per treatment were used in each facility, and IR treatments were applied weekly via foliar sprays. Control plants are represented in grey, phosphite-treated plants in blue, and BABA-treated plants in green. Shaded bands represent the 95% confidence intervals of the GAMM fits and indicate periods of significant differences.

#### 3.1.2 Extracted plant canopy traits following IR treatments over the course of the experiment

Plant canopy area was extracted from top-view RGB images acquired at NaPPI throughout the experiment (Fig. 3A). To assess treatment effects relative to the control, pointwise estimated differences were calculated, and treated conditions were considered to significantly diverge from the control when their 95% confidence intervals did not include zero. At the beginning of the experiment, BABA and KPhi-treated plants showed no significant differences in canopy area compared to the control (Fig. 3B and C). However, from 20 to 35 DAT the canopy area of BABA-treated plants was significantly smaller than that of the control (Fig. 3C), confirmed over a narrower window (22-32 DAT) by a stricter simultaneous-CI test. In contrast, KPhi-treated plants had a significantly larger canopy area than the control during the mid-stage of growth (from 22 to 25 DAT; Fig. 3B), followed by a smaller canopy area toward the end of the time course, with significance observed briefly from 32 to 35 DAT (Fig. 3B), though this KPhi pattern was not confirmed by the simultaneous-CI test. Thus, BABA-treated plants were simultaneously taller and had a smaller lateral canopy compared to controls.

**Fig. 3:**
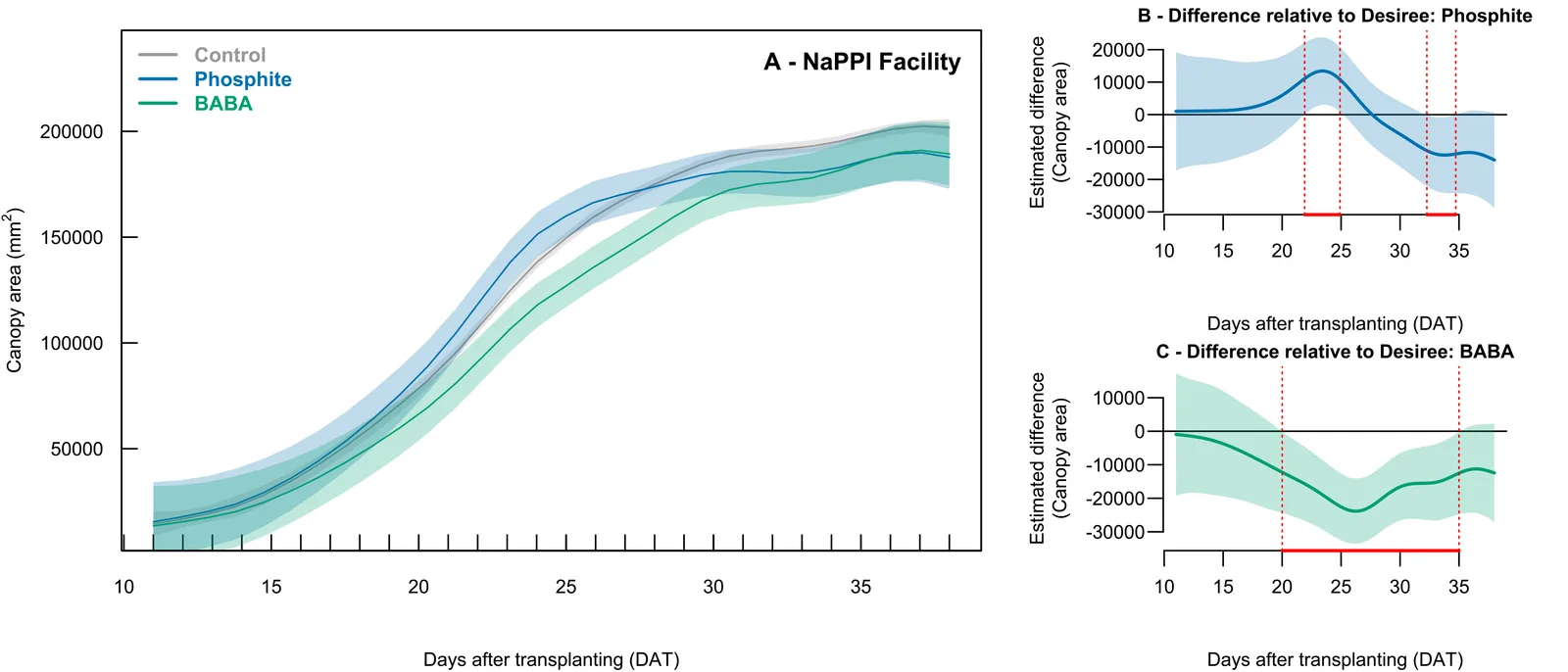
Canopy area dynamics in response to induced-resistance treatments. Generalized Additive Mixed Models (GAMMs) were used to analyse canopy area measured in NaPPI (A). Pointwise differences in the fitted canopy area of phosphite treated plants (B), or BABA treated plants (C), relative to the Désirée (cv.) control, are shown across days after transplanting (DAT), plotted as treatment minus control (positive values indicate a higher value in the treated plants). Group differences were evaluated using two sided pairwise smooth difference tests derived from the GAMMs, with significance concluded when the 95% confidence interval of the difference did not include zero. Six biological replicates per treatment were used, and IR treatments were applied weekly via foliar sprays. Control plants are shown in grey, BABA treated plants in green, and phosphite treated plants in blue. Shaded bands represent the 95% confidence intervals of the GAM fits. Red lines at the bottom of panels B and C indicate periods during which significant differences at the 95% confidence level were detected.

#### 3.1.3 Colour composition following IR treatments over the course of the experiment

The greenness index was calculated from top-view RGB images at NaPPI and single-channel Red, Green, and Blue images at PhenoLab. Greenness profiles of BABA-treated plants were consistently and significantly higher than those of the control from 23 to 38 DAT in NaPPI (Fig. 4B) and from 21 to 34 DAT in PhenoLab (Fig. 4D), whereas KPhi-treated plants differed significantly from the control at NaPPI only briefly, being higher from 33 to 34 DAT (Fig. 4A), a difference not confirmed by the simultaneous-CI test; at PhenoLab, KPhi-treated plants were significantly lower than the control during an early window (approximately 1-3 DAT) and significantly higher during a mid-course window (approximately 14-18 DAT), with no significant difference outside these windows (Fig. 4C), though neither window survives a stricter simultaneous-CI test (Supplementary Table S4). The higher greenness index in BABA-treated plants corresponded to an overall paler canopy colour compared to the control. This apparent contrast arises because the GREENNESS index (Eq. (1)) can increase from a shift in the balance among the red, green and blue channels rather than from a rise in green reflectance alone, and does not necessarily indicate a visually “greener” canopy.

**Fig. 4:**
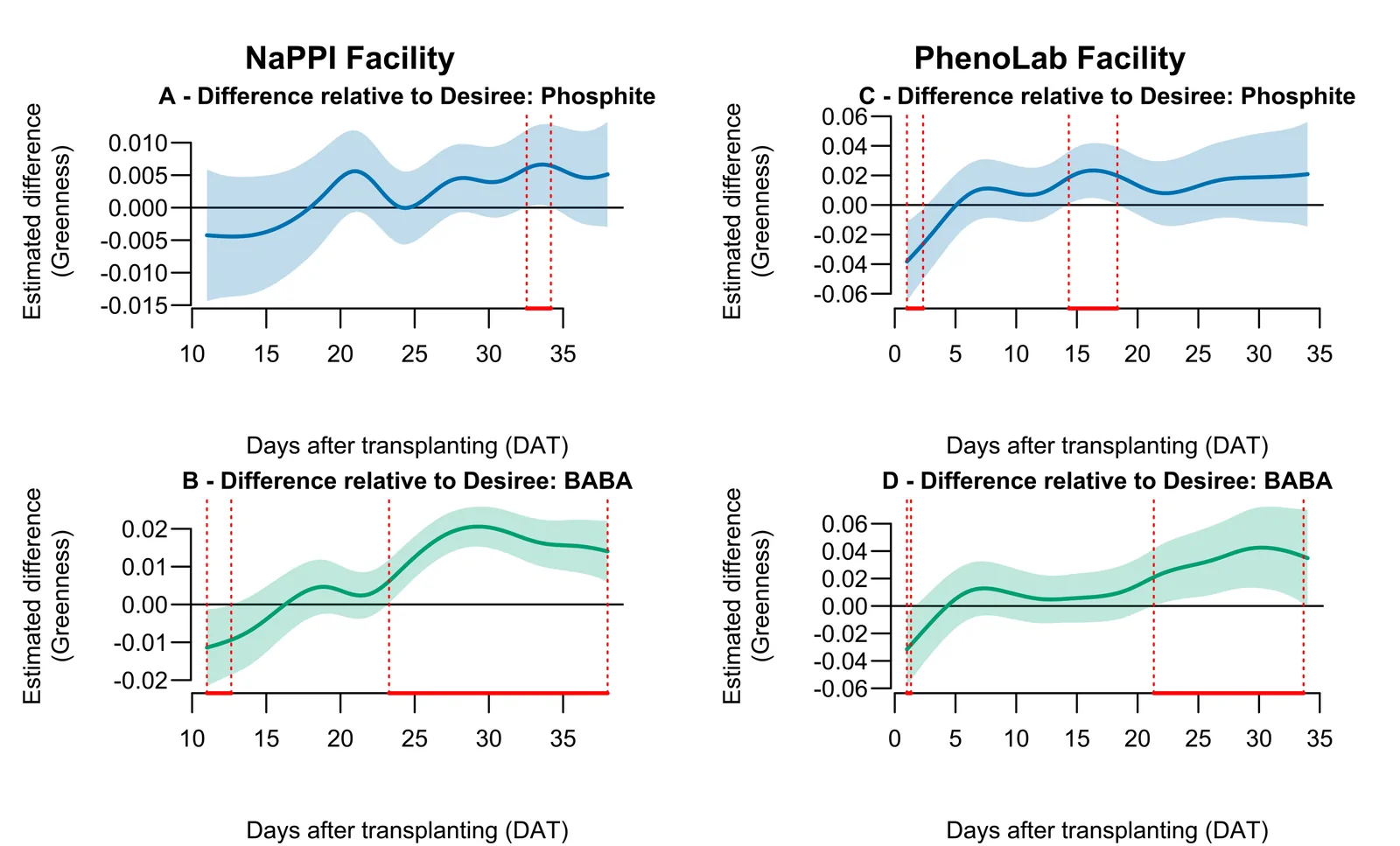
Greenness dynamics in response to induced-resistance treatments. Pointwise differences in greenness of phosphite-treated plants in NaPPI (A) or PhenoLab (C), and of BABA-treated plants in NaPPI (B) or PhenoLab (D), relative to the Désirée (cv.) control, are shown across days after transplanting (DAT), plotted as treatment minus control (positive values indicate a higher value in the treated plants). Group differences were evaluated using two-sided pairwise smooth-difference tests derived from the GAMMs, with significance concluded when the 95% confidence interval of the difference did not include zero. Six biological replicates per treatment were used, and IR treatments were applied weekly via foliar sprays. BABA-treated plants are shown in green, and phosphite-treated plants in blue. Red lines at the bottom of panels A–D indicate periods during which significant differences at the 95% confidence level were detected.

#### 3.1.4 Chlorophyll fluorescence measurements following IR treatments at the end of the experiment

Chlorophyll fluorescence was measured at the end of the experiment at NaPPI. Chlorophyll fluorescence measurements showed no significant differences between control and IR treatments for maximum PSII quantum yield (F_v_/F_m_; Control: 0.827 ± 0.008, BABA: 0.821 ± 0.004, KPhi: 0.824 ± 0.002) or nonphotochemical quenching at light steady state (NPQ_Lss; Control: 0.460 ± 0.040, BABA: 0.493 ± 0.013, KPhi: 0.476 ± 0.005). KPhi-treated plants showed a significant increase in effective PSII quantum yield at light steady state (QY_Lss; 0.602 ± 0.003 vs. control 0.594 ± 0.009) (estimate 0.0083, 95% CI 0.0001 to 0.0166, adjusted p = 0.049) compared to the control, while BABA had no significant effect on QY_Lss (estimate −0.0061, 95% CI -0.0144 to 0.0022, adjusted p = 0.177).

The fluorescence decline ratio (Rfd_Lss), used as an indicator of plant vitality, was significantly influenced by the treatments. BABA-treated plants exhibited lower Rfd_Lss values (estimate −0.083, 95% CI -0.135 to −0.031, adjusted p = 0.0025) whereas KPhi-treated plants showed significantly higher values (estimate +0.098, 95% CI 0.046 to 0.151, adjusted p < 0.001) compared to the control (Fig. 5), indicating a contrasting physiological response between the two inducers.

**Fig. 5:**
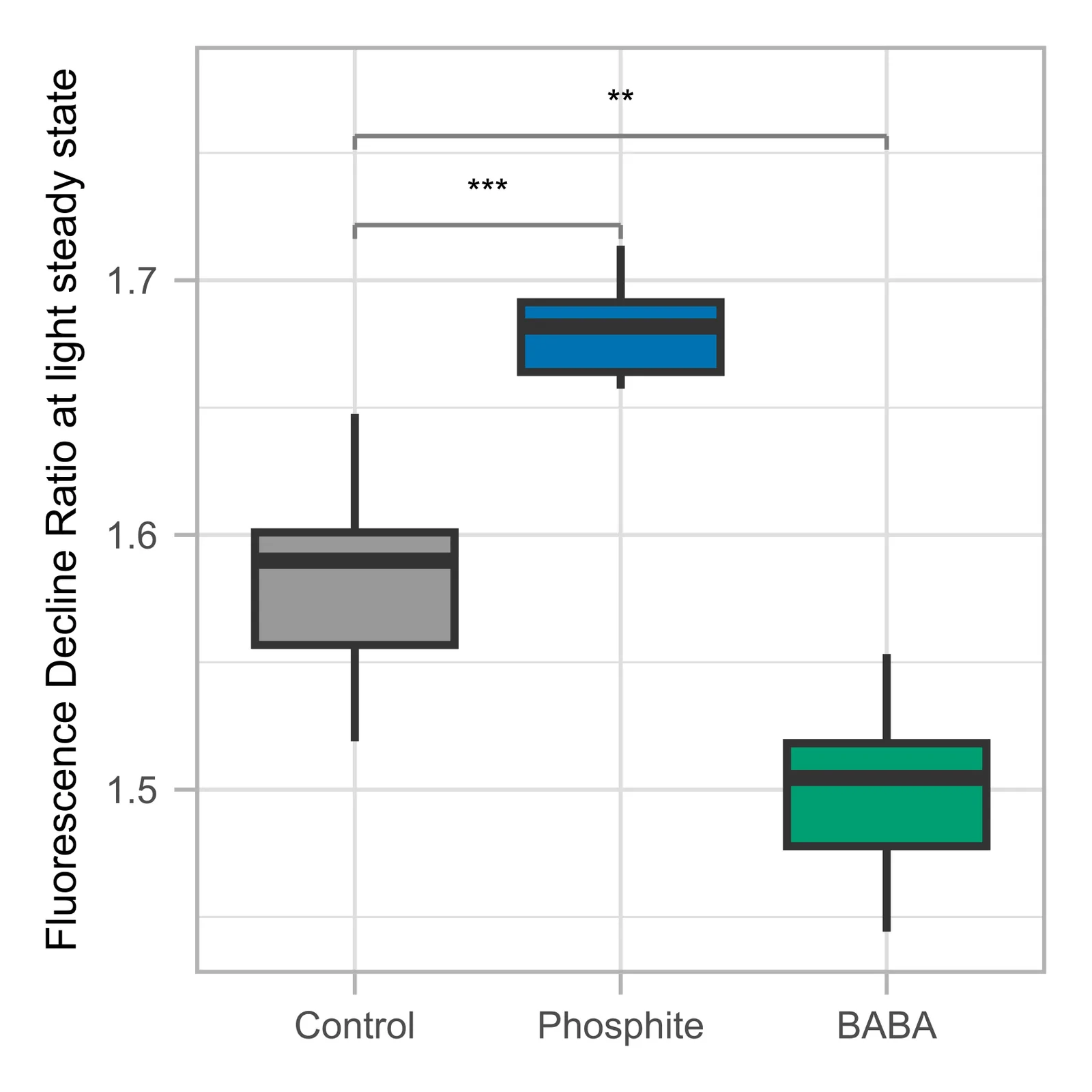
Rfd response to induced-resistance treatments at the end of the treatment. Boxplots show the distribution of the Fluorescence Decline Ratio at light steady state (Rfd_Lss) measured at 34–36 DAT in NaPPI. Six biological replicates per treatment were used. Group differences were assessed using two-sided Dunnett-type multiple-comparison tests applied to estimated marginal means (EMMs). Control plants are shown in grey, phosphite-treated plants in blue, and BABA-treated plants in green. Significant differences between IR treatments and the control are indicated by asterisks above each boxplot (∗∗p < 0.01, ∗∗∗p < 0.001).

#### 3.1.5 Continuous multispectral imaging following IR treatments

Multispectral imaging conducted in PhenoLab enabled calculation of the Normalized Difference Vegetation Index (NDVI), a spectral index commonly used as an indicator of canopy greenness and photosynthetic activity. Both BABA- and phosphite-treated plants maintained significantly higher NDVI values than the control during the early growth stage (Supplementary Fig. S1A, panels B and C). Phosphite-treated plants did not differ significantly from the control thereafter, apart from a brief late window at 32-33 DAT (Supplementary Fig. S1B). BABA-treated plants, however, showed a further significant dip below the control during the mid-course of the experiment (approximately 16-21 DAT), which was not confirmed by the simultaneous-CI test, before returning to values not significantly different from the control for the remainder of the experiment (Supplementary Fig. S1C).

#### 3.1.6 Enzymatic analysis following IR treatments

At the end of the experiment, the activities of eight antioxidant enzymes and twelve carbohydrate-metabolising enzymes were quantified in both facilities (Table S1), giving a single ∼38 DAT endpoint measurement per enzyme rather than a time-resolved trajectory; enzyme-level interpretations below therefore describe a metabolic state consistent with a given process, not a directly observed dynamic process. Their contribution to treatment responses was evaluated using Principal Component Analysis (PCA), treated here as an exploratory analysis, since each treatment is represented by only two or three biological replicates. Tested individually, 13 of the 20 enzymes at NaPPI and 11 at PhenoLab differed significantly among the three treatments after Benjamini-Hochberg correction (Table S1). Treatments formed distinct clusters on the PCA plots in both facilities, indicating that enzymatic profiles were strongly associated with treatment effects (Fig. 6A), though the proportion of variance captured by the first two components differed substantially between facilities (PC1 = 46.7% at NaPPI vs. 69.5% at PhenoLab; total 75.3% vs. 87.8%), reflecting genuinely different metabolic variance architecture. However, in NaPPI, variance in the control group was primarily associated with FK and AGPase, whereas in PhenoLab it was linked to CAT and PGM. For BABA-treated plants, NaPPI showed strong associations with PFK and PGM, while PhenoLab was linked to cwInv. In KPhi-treated plants, NaPPI was associated with HKX and cwInv, whereas PhenoLab correlated with PFK and Susy. Some of these associations (FK and AGPase at NaPPI; PFK at PhenoLab) involve enzymes that did not differ significantly among treatments when tested individually (Table S1); they describe multivariate patterns in the biplot rather than individually significant differences.

**Fig. 6:**
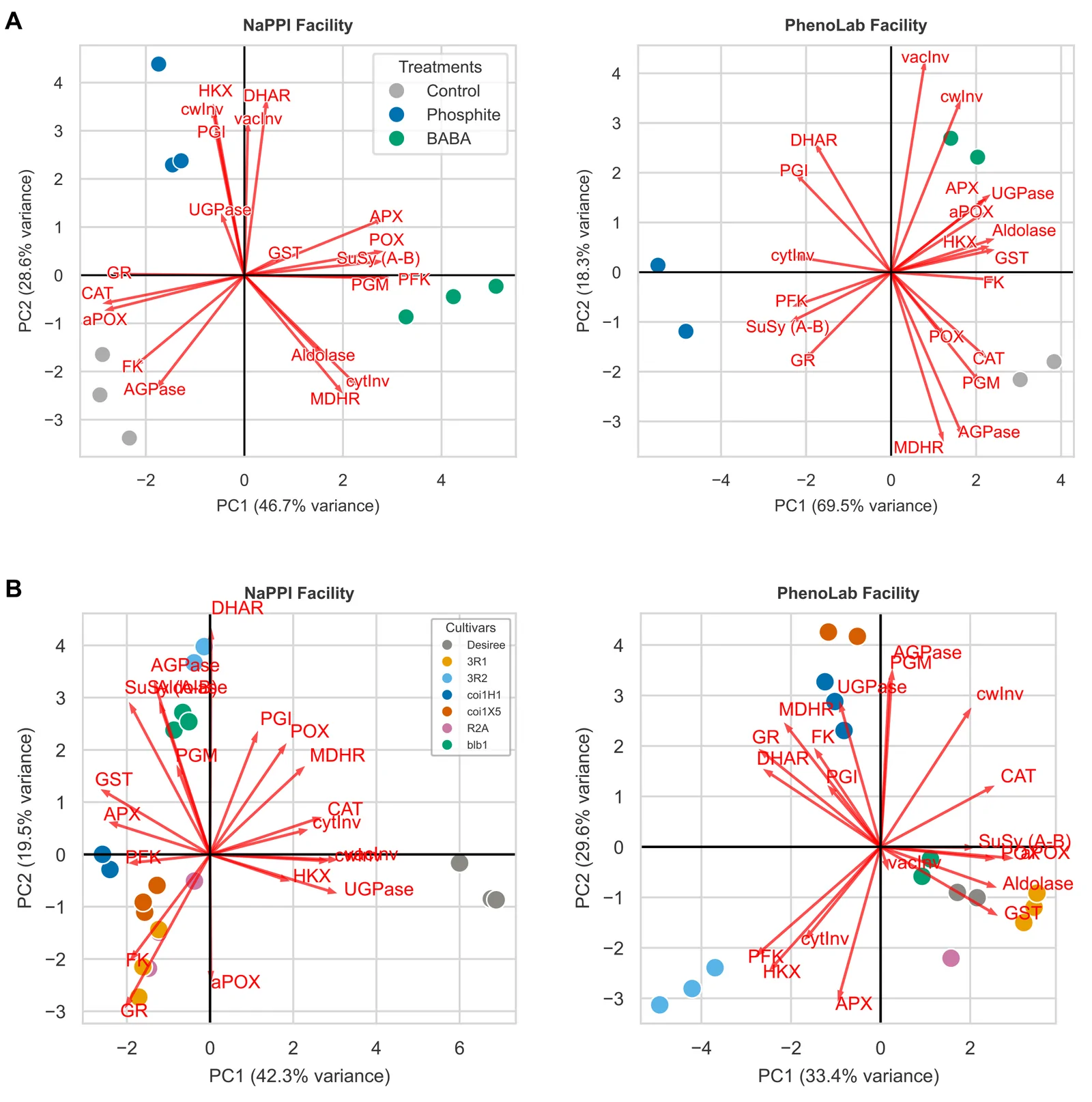
Principal Component Analysis (PCA) of enzymatic profiles in response to induced-resistance treatments and transgenic lines. Two-dimensional PCA scatter plots showing PC1 and PC2 based on endpoint enzymatic profiling from NaPPI (left panels) and PhenoLab (right panels). Induced-resistance treatments (A) include three biological replicates per treatment in NaPPI and two replicates in PhenoLab. Control plants are shown in grey, phosphite-treated plants in blue, and BABA-treated plants in green. Transgenic lines (B) include three biological replicates per line in both facilities. Control plants are shown in grey; transgenic lines are colour-coded as follows: 3R1 (amber), 3R2 (pale blue), coi1H1 (dark blue), coi1X5 (orange), R2A (pink), and blb1 (green). In all panels, red arrows indicate loading vectors representing the contribution and direction of each measured enzyme in the biplot. PC1 and PC2 together explained 75.3% (NaPPI) and 87.8% (PhenoLab) of the total variance for the induced-resistance treatments (A; PC1 = 46.7% and 69.5%, respectively), and 61.8% (NaPPI) and 63.0% (PhenoLab) for the transgenic lines (B; PC1 = 42.3% and 33.4%, respectively), reflecting genuinely different metabolic variance architecture between facilities.

A key feature of the enzymatic PCA is the consistent divergence between BABA and KPhi in the enzyme classes driving their respective separations from controls. Within each facility, the two inducers were associated with distinct endpoint enzyme signatures (Fig. 6A). The identity of the specific enzymes driving BABA and KPhi separation differed between facilities; with data from two experiments, we cannot attribute this variation to a specific environmental factor.

In summary, the enzyme activity profiling revealed that in both facilities the IR treatments resulted in distinct signatures, indicating effects on both primary carbohydrate metabolism and antioxidant metabolism versus the mock controls. However, the key determinants of the differential responses were distinctly different in the two facilities. This was reflected in specific clustering when the data from both facilities were jointly analysed in a single PCA (Supplementary Fig. S2).

#### 3.1.7 End point measures of biomass following IR treatments

Final above-ground and tuber measurements were taken in both facilities at the end of the experiment. BABA-treated plants in NaPPI showed a trend toward lower fresh weight than controls, a pattern not recorded in PhenoLab (Supplementary Fig. S3, upper panels). In contrast, dry weight in NaPPI was significantly reduced in these plants (estimate −3.87 g, 95% CI -7.23 to −0.51, adjusted p = 0.024), while PhenoLab exhibited a similar but non-significant trend (Fig. 7). This pattern was opposite to the results for plant height but aligned with those for canopy area. No significant differences from the control were observed for KPhi-treated plants in either facility for both fresh and dry weight (Supplementary Fig. S3 and Fig. 7), which was consistent with other growth measurements. Differences in plant growth did not translate into significant changes in number of tubers or tuber weight compared to the control in either facility (Supplementary Fig. S3), though tubers were assessed at an early stage of filling (∼38 DAT; plants about 7.5 to 8.5 weeks old) and this absence of a detectable difference should not be read as evidence that no such difference exists at a later harvest stage. Nonetheless, a common pattern was observed in both facilities, with tuber number tending to be higher in KPhi-treated plants in NaPPI and PhenoLab (Supplementary Fig. S3). Full numerical results (sample size, mean, SD, effect estimate, 95% CI, and adjusted p-value) for all above-ground and tuber endpoint comparisons, at both facilities, are given in Supplementary Table S3.

**Fig. 7:**
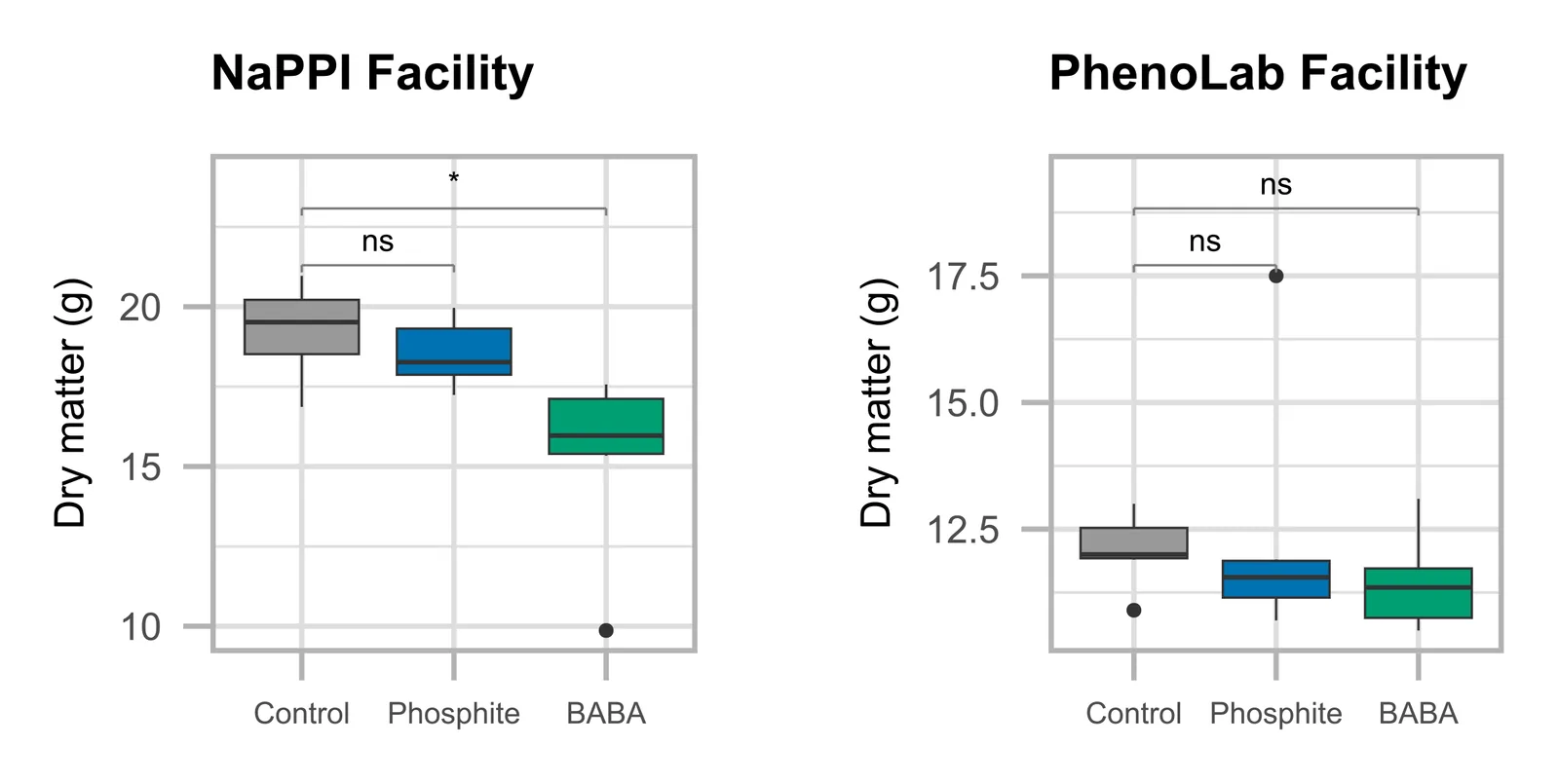
Dry matter response to induced-resistance treatments. Boxplots showing above-ground dry matter in NaPPI (left panel) and PhenoLab (right panel) at the end of the experiment. Each treatment group includes six biological replicates, except the NaPPI Désirée/Control group (n = 4; see Supplementary Table S3). Group differences were assessed using two-sided Dunnett-type multiple-comparison tests applied to estimated marginal means (EMMs). A significant difference between an IR treatment and the control is indicated by an asterisk (∗p < 0.05); ns indicates no significant difference.

### 3.2 Transgenic lines with different resistance genes

Six transgenic lines in the Désirée background (Table 1) were evaluated across both facilities over four weeks: R2A, blb1, 3R1, 3R2, coi1H1, and coi1X5.

#### 3.2.1 Plant height in transgenic lines over the course of the experiment

To assess treatment effects on plant height relative to the control, pointwise estimated differences were used. Lines expressing a single *Rpi* gene, R2A, showed no clear difference compared to the control in either facility (Supplementary Fig. S4A and C, top panels), whereas lines expressing the single *Rpi* gene blb1 exhibited significantly lower plant height in NaPPI, briefly during early growth and continuously from 28 to 38 DAT, confirmed from 31 to 34 DAT by a stricter simultaneous-CI test, and sustained lower height from 17 to 34 DAT in PhenoLab (Supplementary Fig. S4B and D), confirmed over a slightly narrower window (18-34 DAT) by the same test.

In NaPPI, lines with stacked *Rpi* genes showed contrasting patterns: 3R1 was significantly taller than the control only at the very end of the experiment, from 36 to 38 DAT, a difference not confirmed by the simultaneous-CI test, while 3R2 was significantly taller than the control from 34 to 38 DAT (Fig. 8A, top panels), confirmed over a narrower window (35-38 DAT) by a simultaneous-CI test. Both the coi1 lines H1 and X5, which have reduced sensitivity to jasmonic acid methyl ester, were taller at the end of the experiment, with significant differences from 34 to 38 DAT (Fig. 8A, bottom panels), a difference the simultaneous-CI test confirmed for both lines over narrower windows, from 36 to 38 DAT for coi1H1 and from 37 to 38 DAT for coi1X5. The same patterns were observed in PhenoLab, confirming these observations (Supplementary Fig. S5), where all significant genotype plant-height comparisons remained significant, over narrower windows, under the simultaneous-CI test.

**Fig. 8:**
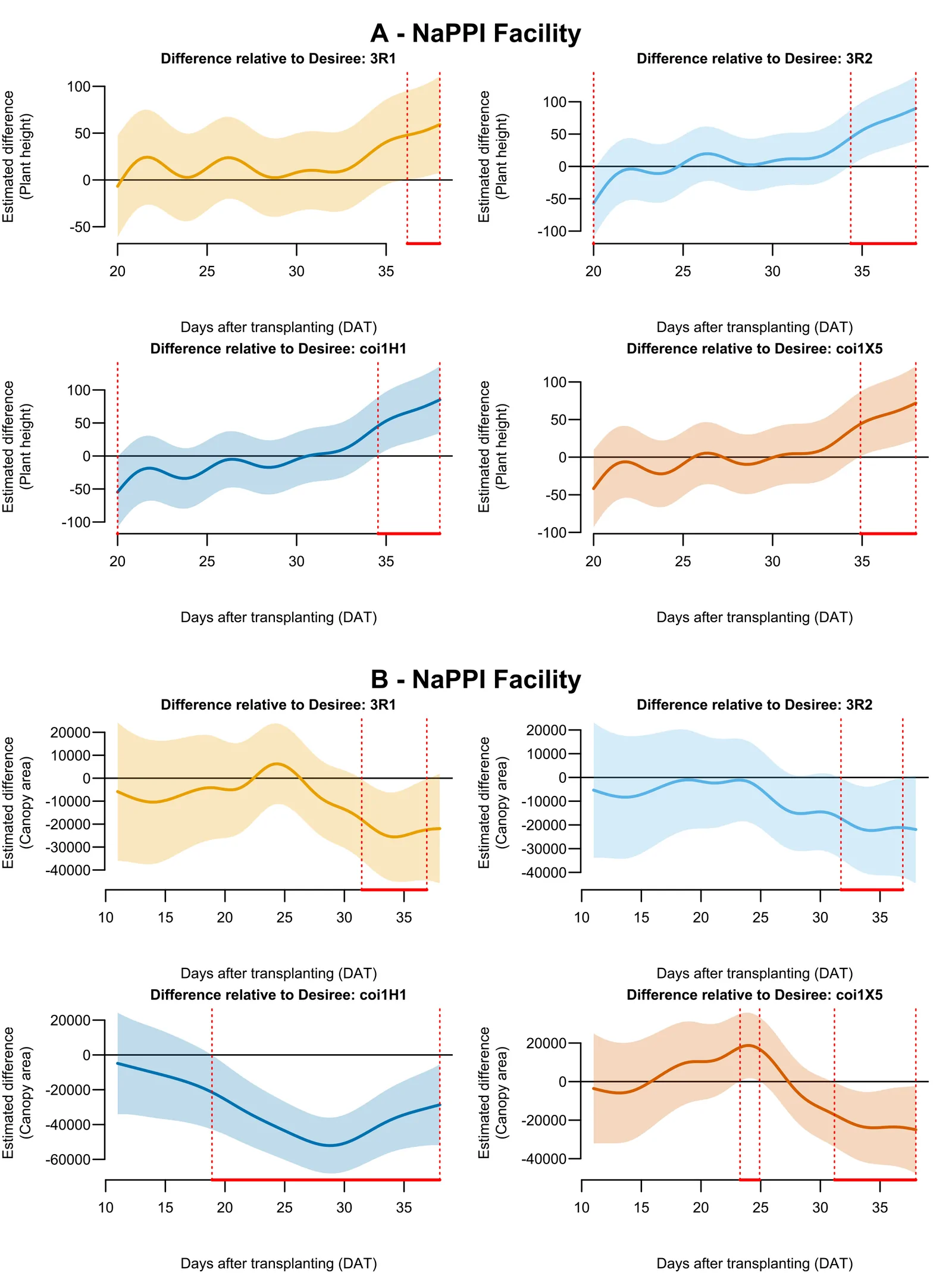
Plant height and canopy area dynamics in NaPPI for transgenic lines containing stacked Rpi genes (3R1, 3R2) or modified phytohormone pathways (coi1H1, coi1X5). Pointwise differences in plant height (A) and canopy area (B) of the transgenic lines 3R1 (amber), 3R2 (pale blue), coi1H1 (dark blue), and coi1X5 (orange) relative to Désirée (cv.) are shown across days after transplanting (DAT), plotted as transgenic line minus Désirée (positive values indicate a higher value in the line). Group differences were evaluated using two-sided pairwise smooth-difference tests derived from the generalized additive mixed models (GAMMs), with significance concluded when the 95% confidence interval of the difference did not include zero. Six biological replicates per genotype were used. Red lines at the bottom of the plots indicate periods during which significant differences at the 95% confidence level were detected.

#### 3.2.2 Extracted plant canopy traits in transgenic lines over the course of the experiment

Total canopy area was measured for transgenic lines in NaPPI (Fig. 8B). The single *Rpi* gene line R2A showed no clear difference compared to the control, whereas blb1 had a significantly lower canopy area from 29 to 34 DAT, not confirmed by the simultaneous-CI test (Supplementary Fig. S6). The coi1H1 line exhibited a significantly lower canopy compared to control from 19 DAT until end of the growth period (Fig. 8B, bottom left panel), confirmed over a narrower window (21-37 DAT) by a simultaneous-CI test. The coi1X5 line, however, showed a short significant higher canopy during the mid-course (from 23 to 25 DAT), followed by a significant lower canopy toward the end of the experiment (from 31 to 38 DAT; Fig. 8B, bottom right panel), though neither difference was confirmed by the simultaneous-CI test. Other lines with stacked *Rpi* genes, 3R1 and 3R2, displayed a comparable canopy trajectory, with canopy area higher than the control during the mid-course and significantly lower toward the end of the experiment (from 31 to 37 DAT for both lines; Fig. 8B, upper panels), though neither late-course reduction was confirmed by the simultaneous-CI test.

#### 3.2.3 Colour composition and chlorophyll fluorescence measurements in transgenic lines

The greenness index measured in transgenic lines showed small, intermittent significant differences from the control at both facilities, mostly confined to brief early- or mid-course windows. The exceptions were the coi1 lines. At PhenoLab, coi1H1 showed a sustained, significantly higher greenness index for much of the growth period, confirmed from 12 to 20 DAT by a stricter simultaneous-CI test. At NaPPI, both coi1 lines showed a higher greenness index from about 31 DAT to the end of the experiment, confirmed from 33 to 35 DAT by the simultaneous-CI test for coi1X5 (Supplementary Table S4). Similarly, chlorophyll fluorescence parameters measured in NaPPI, F_v_/F_m_, QY_Lss, and NPQ_Lss, did not differ significantly from control. The plant vitality indicator Rfd_Lss in the coi1 mutant lines (H1 and X5) did not differ significantly from the control (Supplementary Fig. S7). In contrast, Rfd_Lss tended to be slightly lower in lines carrying stacked Rpi genes (3R1 and 3R2) as well as in lines containing a single Rpi gene (R2A and blb1); however, these differences were not statistically significant (Supplementary Fig. S7).

#### 3.2.4 Continuous multispectral imaging of transgenic lines

NDVI measurements in PhenoLab showed that all transgenic lines increased until reaching a plateau, from approximately 0.82 at early stages to 0.87 by mid-experiment. Pointwise comparisons between each transgenic line and the control revealed that lines carrying stacked Rpi genes (3R1, 3R2) and those with modified phytohormone pathways (coi1H1, coi1X5) exhibited significantly higher NDVI values than the control during the early growth stage (Supplementary Fig. S8). Among single Rpi gene lines, neither R2A nor blb1 displayed early NDVI elevation; blb1 showed no significant NDVI difference at any stage, while R2A exhibited a significant elevation relative to the control during the later growth stage (Supplementary Fig. S8). This grouping was observed at PhenoLab only; whether it reflects a common underlying mechanism is an open question, given that stacked Rpi lines constitutively activate NLR-mediated immunity while coi1 lines have disrupted JA signalling.

#### 3.2.5 Enzymatic analysis in transgenic lines

Principal Component Analysis (PCA) was performed to evaluate the contribution of enzyme activities to variation among transgenic lines across the two facilities (Fig. 6B), treated here as an exploratory analysis for the same reason as above. As with the induced-resistance treatments, the proportion of variance captured by the first two components again differed substantially between facilities (PC1 = 42.3% at NaPPI vs. 33.4% at PhenoLab; total 61.8% vs. 63.0%). Tested individually, all 20 enzymes differed significantly among the seven genotypes at both facilities after Benjamini-Hochberg correction (Table S1). In NaPPI, control samples clustered opposite to most transgenic lines, primarily associated with high loadings of Invertase and UGPase. Lines carrying stacked Rpi genes (3R1, 3R2) segregated in distinct directions: variance in 3R1 was mainly explained by FK and GR, whereas 3R2 was associated with DHAR. Transgenic lines with modified phytohormone pathways (coi1H1, coi1X5) clustered closely together, strongly correlated with PFK. Single Rpi gene lines (R2A, blb1) showed contrasting patterns: R2A grouped near 3R1, while blb1 clustered with 3R2 (Fig. 6B, left panel).

In PhenoLab, clustering patterns differed markedly. Control samples grouped together with blb1 and 3R1, associated with Aldolase and GST. The other stacked Rpi line (3R2) formed a distinct cluster linked to PFK and HKX, while R2A also separated independently. Both coi lines (coi1H1, coi1X5) again clustered together, associated with UGPase and AGPase (Fig. 6B, right panel).

The coi1H1 and coi1X5 lines produced the most internally coherent enzymatic clustering of any genotype in this study, co-clustering tightly in both facilities (Fig. 6B). However, the metabolic drivers of this clustering differed entirely: PFK dominated at NaPPI, whereas AGPase, UGPase, and PGM drove the separation at PhenoLab.

In summary, the enzyme activity profile revealed that in both facilities the different transgene-mediated resistance resulted in distinct biosignatures, indicating differential effects on both primary carbohydrate metabolism and antioxidant metabolism in relation to the mock control. However, the key determinants of the differential responses were completely different in the two facilities. This was reflected in a distinct, facility-associated clustering when the data from both facilities were jointly analysed in a single PCA (Supplementary Fig. S2).

The combined cross-facility PCA (Supplementary Fig. S2) shows facility-associated clustering, with NaPPI and PhenoLab control plants occupying distinct metabolic positions. Control plants in NaPPI were associated with sucrose-mobilising enzymes (UGPase, vacInv), while PhenoLab controls were associated with glycolytic and sucrose synthase activities (FK, SuSy). The available data do not allow attribution of these baseline differences to a specific environmental factor.

#### 3.2.6 End point measures of biomass in transgenic lines

Across both facilities, patterns of biomass and yield responses varied by genotype and environment (Fig. 9 and S9). In NaPPI, most transgenic lines exhibited lower fresh and dry biomass compared to the control, although these differences were generally not statistically significant, except for coi1X5, which showed a significantly lower dry weight (Fig. 9 left panel). Regarding yield, 3R1 produced a significantly greater number of tubers, and coi1X5 had a significantly higher tuber weight, while all other lines showed no significant differences in tuber number or weight compared to the control (Supplementary Fig. S9 middle and bottom panels of the left side); as tubers were assessed at an early stage of filling (∼38 DAT; plants about 7.5 to 8.5 weeks old), these tuber differences are indicative only. In PhenoLab, significant reductions in biomass were observed for lines carrying stacked Rpi genes (3R1, 3R2) and single Rpi genes (R2A, blb1), with fresh weight significantly reduced for 3R1, 3R2, and blb1, and dry weight significantly reduced for all four lines. On the contrary, coi1H1 displayed a significant increase in both fresh and dry weight, while coi1X5 showed a non-significant trend toward increased dry weight (Fig. 9 right panel). Tuber number tended to be higher across transgenic lines, but only 3R2 and R2A were significantly greater than the control, a difference that is likewise indicative only, given the early harvest and the incomplete tuber records at PhenoLab. Tuber weight varied among lines, higher in 3R1 and coi1X5 and lower in coi1H1, blb1, 3R2, and R2A, but none of these differences were statistically significant (Supplementary Fig. S9, bottom right panel). However, for most lines, tuber number and weight did not differ significantly from the control, with the notable exception of coi1X5, which produced higher tuber biomass in NaPPI. At PhenoLab, tuber number and tuber weight records were incomplete for five of the six transgenic lines (3R1, n = 1; coi1H1, n = 3; blb1 and coi1X5, n = 4; 3R2, n = 5), while R2A retained six; above-ground fresh and dry weight were unaffected at PhenoLab (six for all groups). Full numerical results are given in Supplementary Table S3; the NaPPI genotype comparisons for tuber number and tuber weight used a linear mixed model with Block as a random intercept, which for these two traits gave estimates of +8.6 tubers (95% CI 0.7 to 16.5, adjusted p = 0.026) for 3R1 and +50.2 g (95% CI 10.5 to 89.9, adjusted p = 0.007) for coi1X5, consistent with the pattern described above. Tuber counts and weights at both facilities should be interpreted with caution given the early harvest relative to each facility's pot volume, especially at PhenoLab, where pots were smaller.

**Fig. 9:**
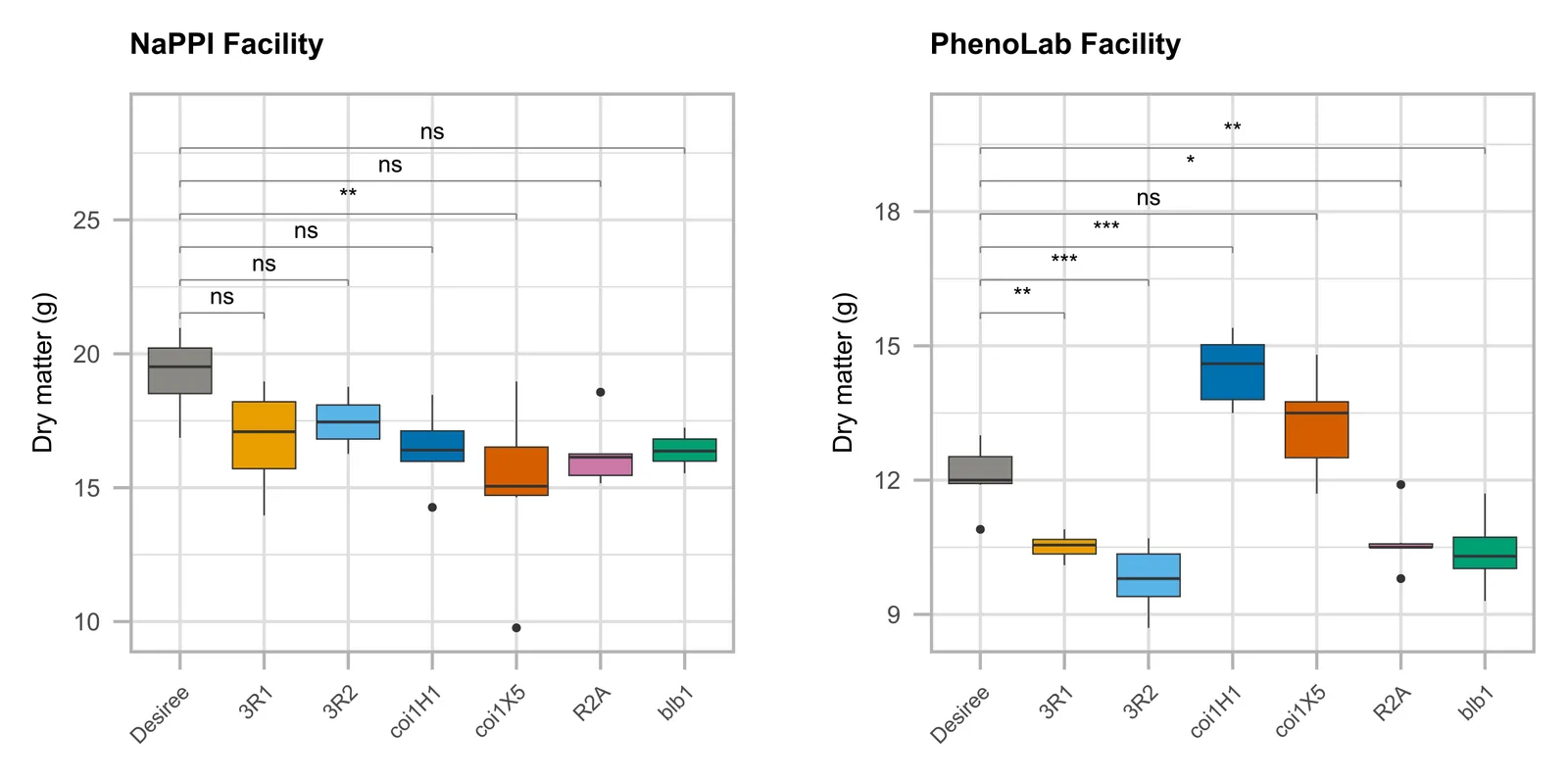
Dry matter response in transgenic lines containing stacked Rpi genes (3R1, 3R2), modified phytohormone pathways (coi1H1, coi1X5), and single Rpi genes (R2A, blb1). Boxplots showing above-ground dry matter in NaPPI (left panel) and PhenoLab (right panel) at the end of the experiment. Each genotype includes six biological replicates, except the shared Désirée/Control group at NaPPI (n = 4; see Supplementary Table S3). Group differences were assessed using two-sided Dunnett-type multiple-comparison tests applied to estimated marginal means (EMMs). Significant differences between transgenic lines and the control are indicated by asterisks: ∗p < 0.05, ∗∗p < 0.01, ∗∗∗p < 0.001; ns indicates no significant difference.

## 4 Discussion

Recent advances in sensor-based imaging and automation technology allow high-throughput phenotyping for multiple traits related to plant development under different stress conditions. Its application to assessing the allocation responses of plant defence strategies, however, remains limited. This study examines how potato plants allocate resources between growth and defence, using chemical resistance inducers and transgenic lines with distinct resistance mechanisms. A two-facility design was used to distinguish consistent allocation responses from those that vary with growing conditions, across traits ranging from morphological to metabolic. We used image-based sensors to track growth and morpho-physiological changes over time in the absence of the pathogen. We found morphological and physiological changes in response to IR treatment and in the transgenic lines, suggesting that these phenotyping methods may be useful for investigating potential allocation responses triggered by resistance inducers or resistance genes.

To activate disease resistance in *Arabidopsis*, the biologically active R-enantiomer of BABA binds to the aspartyl tRNA synthetase (AspRS) IBI1, resulting in the accumulation of uncharged tRNA^Asp^ and, consequently, plant stress and growth reduction occurs if BABA is applied at high concentrations [63]. We used a BABA concentration of 10 mM, previously shown to be effective against potato late blight, and at this concentration changes in transcript expression and protein secretion in potato plants have been documented [23], [64]. After four weekly BABA treatments, we observed boosted plant height in both facilities and reductions in canopy area and dry matter at NaPPI. These results are consistent with observations in *Arabidopsis thaliana* where BABA-IR shifted to a BABA-induced stress status, hypothesised to be linked to the concentrations used [25]. The combination of increased plant height, consistently observed in both facilities, alongside reduced canopy area and dry matter at NaPPI, is not consistent with uniform growth suppression but rather with a reallocation of growth resources toward vertical elongation at the expense of lateral canopy expansion [33]. Notably, this above-ground allocation cost did not translate to a reduction in tuber number or weight in our study, where tubers were assessed at early stages of filling, suggesting that below-ground sink strength was maintained against foliar defence activation under these conditions; whether this buffering extends to later harvest stages or to field-grown plants with unconstrained root systems remains to be tested. The paler canopy colour recorded by the greenness index at both facilities, alongside higher early NDVI values at PhenoLab, is consistent with altered pigment ratios or modified leaf optical properties rather than reduced chlorophyll content per se, a distinction relevant for interpreting BABA's effect on canopy physiology. The early NDVI elevation shared by both BABA and KPhi at PhenoLab, preceding their morphological divergence, may reflect an initial common canopy response before the differential allocation costs of the two inducers become apparent over repeated treatments. At the cell-physiological level, the endpoint enzyme profiles are consistent with a defence-associated carbon-reallocation signature in BABA-treated plants. BABA-treated plants showed enzymatic signatures associated with defence-related carbon reprogramming, with elevated glycolytic enzyme activity at NaPPI and elevated invertase-linked, sucrose-mobilising enzyme activity at PhenoLab, signatures well-documented as components of such reprogramming in other systems [27], [28], [65]. The Rfd_Lss reduction at NaPPI is consistent with reduced CO2 fixation capacity, pointing to carbon-limited assimilation rather than PSII damage per se [49], [66]. Together, these layers of evidence (morphological, enzymatic at both facilities, and fluorometric at NaPPI) converge on a model in which BABA is associated with architectural reallocation and an endpoint carbon-metabolism signature consistent with carbon rerouting, rather than generalized growth inhibition.

There is evidence that phosphite applications can influence plant growth and architecture. The impact of phosphite on various aspects of plant development, including morphology, photosynthesis, and resistance to pathogens, has been documented in several studies [67], [68]. We previously showed that phosphite and BABA treatment of potato cv. Désirée at the same amounts as the present study led to a 40% overlap of differentially expressed genes [20]. Despite this transcriptional overlap, the endpoint enzyme profiling presented here reveals that the metabolic signatures associated with KPhi and BABA are consistently distinct from each other within each facility, with KPhi consistently imposing a lower apparent growth cost than BABA in both facilities, although the specific enzymatic identity of each signature was not conserved across facilities. This discordance between transcriptomic overlap and enzyme-level divergence underlines the added value of physiological phenotyping as a complement to molecular profiling in dissecting defence allocation mechanisms. Nevertheless, the repeated treatment of phosphite did not produce morphological differences confirmed by the simultaneous-CI test compared to the untreated control. The absence of a detectable growth penalty at these concentrations is consistent with KPhi's suitability as a plant protection agent, applicable at levels giving protection against *P. infestans* without a detectable cost to plant performance under the conditions tested.

We next examined allocation responses associated with genetically encoded resistance mechanisms, using transgenic lines representing three distinct strategies: single Rpi genes, a stacked three-gene Rpi construct, and RNAi-mediated disruption of jasmonate signalling.

There are few studies where the morphology of resistance genes including *Rpi-blb1* has been studied over time in such detail. We have previously used these specific potato transgenic *Rpi-blb1* lines and did not notice a clear morphological difference [69]. The 3R gene construct was evaluated during the growth season and no statistical difference in morphology was detected between the transgene and its genetic background by weekly measurements in the field [70], suggesting that environmental conditions outweigh genotypic effects on plant morphology. In contrast to these previous findings and to R2A, which showed no height difference in either facility, blb1 showed a significant height reduction at PhenoLab from mid-course to the end of the experiment; at NaPPI, plants were consistently below control and significantly so from 28 DAT to the end of the experiment, a reduction confirmed at both facilities by the simultaneous-CI test. This pattern is consistent with a modest allocation response associated with Rpi-blb1 expression, potentially reflecting constitutive downstream signalling activity even in the absence of pathogen challenge. 3R2 plants were significantly taller than the control at the end of the experiment, a pattern confirmed at both facilities. This height gain in a stacked Rpi line contrasts with the height reduction seen in blb1 and may reflect a hormonal rebalancing effect arising from constitutively expressed, stacked NLR-mediated immunity, though its mechanistic basis remains to be determined. At the canopy level, the stacked Rpi lines (3R1, 3R2) showed a characteristic pattern at NaPPI only: above-control canopy area during mid-course followed by below-control values toward the end, a trajectory resembling that of KPhi-treated plants rather than the more sustained canopy reduction seen in BABA-treated or coi1H1 plants, though neither the 3R1/3R2 nor the KPhi late-course reduction was confirmed by a simultaneous-CI test, so this comparison rests on descriptive trends rather than confirmed effects. Among the Rpi lines, blb1 and R2A showed divergent patterns: blb1 displayed a height reduction at both facilities and reduced biomass at PhenoLab, while R2A showed no height difference at either facility and a significant reduction in dry weight but not fresh weight at PhenoLab. This divergence within the single-gene group limits generalisation across it. Consistent with the absence of field-level morphological penalties reported for 3R constructs [70], the patterns detected here in controlled conditions do not suggest allocation responses of a magnitude that would be expected to affect cultivar performance. At the enzymatic level, individual Rpi lines showed distinct associations at NaPPI: 3R1 and R2A were both associated with the antioxidant enzyme GR, while 3R2 and blb1 co-clustered, associated with DHAR. Both enzymes belong to the ascorbate-glutathione cycle, suggesting constitutive antioxidant activity across multiple lines regardless of gene copy number. The early NDVI elevation at PhenoLab was observed in stacked Rpi and coi1 lines but not in single-gene lines; R2A showed a late-stage elevation absent in blb1, further illustrating the heterogeneity of phenotypic responses within the single-gene group. A shared mechanistic interpretation for the early NDVI grouping remains uncertain given the opposing immune configurations of the stacked Rpi and coi1 groups and the single-facility nature of this observation.

RNAi knockdown of *StCOI1* in lines H1 and X5 reduced sensitivity to jasmonic acid methyl ester, enabling assessment of allocation responses under disrupted hormone-mediated defence. While specific studies detailing the effects of the coi transgene on potato height are lacking, the literature indicates that transgene expressions, particularly those that have the potential to influence hormonal pathways or metabolic processes, can result in dwarfing effects or altered height. In *Arabidopsis thaliana* and *Nicotiana benthamiana*, Noir et al. [71] observed that MeJA inhibits leaf growth through its receptor, COI1, by reducing the number and size of cells, which ultimately affects overall plant height. Hu *et al.* [72] further demonstrated that COI1-mediated JA signalling represses shoot elongation in citrus, providing cross-species evidence for a growth-repressive function of COI1. These observations are extended here to a crop context: the consistent and cross-facility-reproducible height gain in both coi1 lines is consistent with a constitutive role of JA-mediated signalling in restricting stem elongation in potato [73]. The morphological parallels between coi1H1 and BABA-treated plants, both taller with reduced lateral canopy area at NaPPI, suggest that JA disruption and BABA-mediated priming can converge on similar architectural reallocation outcomes despite engaging distinct molecular pathways. coi1X5, by contrast, showed a canopy trajectory at NaPPI more similar to KPhi-treated plants, though as above neither pattern was confirmed by the simultaneous-CI test, indicating that the two coi1 lines diverged at the canopy level despite sharing the same height phenotype, itself confirmed by the simultaneous-CI test for both lines, and enzyme clustering. Importantly, the metabolic enzyme data reveal that this shared morphological phenotype is supported by different carbon metabolism configurations depending on the growing environment, PFK-dominated at NaPPI, AGPase/UGPase-dominated at PhenoLab, illustrating that phenotypic robustness and metabolic degeneracy can coexist in plant growth-defence allocation responses. The PFK-dominated signature at NaPPI is consistent with enhanced glycolytic flux, possibly reflecting relief of JA-mediated restraint on central carbon metabolism [74], while the AGPase/UGPase-dominated signature at PhenoLab points toward shifts in starch-biosynthetic (AGPase) and sucrose-metabolising (UGPase) capacity, likely reflecting the shorter photoperiod (16 h vs. 18 h) and different substrate availability of the autumn experiment.

Chlorophyll fluorescence is commonly affected when plants are under stress [75], [76]. In this study, BABA and phosphite treatments did not significantly alter the maximum quantum efficiency of PSII photochemistry (F_v_/F_m_), a robust photosynthetic efficiency indicator. Small changes in F_v_/F_m_ values were observed over time, but always within limits of those from a healthy plant tissue. The divergent Rfd_Lss response, decreased by BABA, increased by KPhi, provides a sensitive physiological readout that discriminates between the two inducers even when F_v_/F_m_ is unchanged. The reduced Rfd_Lss in BABA plants is best interpreted as evidence of increased excitation pressure under actinic light, consistent with reduced capacity for CO_2_ fixation due to carbon-limited assimilation downstream of a carbon-reallocation response consistent with defence activation [73]. The increased Rfd_Lss under KPhi, alongside a significant increase in effective quantum yield (QY_Lss; 0.602 ± 0.003 vs. control 0.594 ± 0.009), is consistent with a mild enhancement of photosynthetic performance, compatible with reported biostimulant properties of phosphite [67]. Together, these fluorescence data provide a third, independent line of evidence that BABA and KPhi impose qualitatively different physiological states in potato leaves, a conclusion that could not be reached from imaging data alone.

The use of two independent phenotyping platforms, NaPPI and PhenoLab, provides significant methodological value by enabling the systematic benchmarking of high-throughput systems and assessing whether resistance-associated phenotypes recur across two independently confounded growing environments.

This consistency is noteworthy given the differences in environmental control and cultivation conditions between the two facilities. At NaPPI, plants were grown in 5 L pots under slightly warmer, drier conditions with marginally higher light intensity (day averages of 25.6 °C, 37.5% RH; 250 μmol m^−2^ s^−1^), whereas at PhenoLab they were maintained in 2.2 L pots under cooler, more humid conditions with slightly lower light (22.8 °C, 56.9% RH; 224.3 μmol m^−2^ s^−1^). In addition, NaPPI showed greater thermal variability, with daytime temperatures reaching 35.9 °C, while PhenoLab remained more stable, with a maximum of 25.1 °C. Despite these contrasting conditions, the BABA-associated increase in plant height and paler canopy colour, and the KPhi-associated absence of growth penalty, were both reproduced across facilities. Among the transgenic lines, a below-control height in blb1 and the height gain in 3R2 were observed across both facilities. The comparative assessment of the impact of IR treatments and resistance-inducing transgenes on key enzymes of carbohydrate and antioxidant metabolism revealed facility-associated differences in these cell physiological proxies [29], [32]. The enzyme signatures were not consistent between facilities. The pattern of inconsistency is itself informative: at NaPPI, BABA plants were associated with glycolytic enzymes (PFK, PGM) while KPhi was associated with invertase-related enzymes (HKX, cwInv); at PhenoLab, this assignment reversed, with BABA associated with cwInv and KPhi with PFK and SuSy. The unifying principle is that BABA and KPhi were consistently associated with contrasting endpoint enzyme signatures within each facility, while the identity of the enzymes involved was not conserved across facilities. The contrasting seasonal conditions (May–June at NaPPI, late October to early December at PhenoLab) are known to influence primary carbon metabolism. A combined cross-facility PCA confirmed this facility-associated clustering, with NaPPI and PhenoLab control plants occupying distinct metabolic positions. Specifically, NaPPI control plants were associated with sucrose-mobilising enzymes (UGPase, vacInv), while PhenoLab controls were associated with glycolytic and sucrose synthase activities (FK, SuSy). The growing conditions at PhenoLab from late October to early December, where LED supplementation maintained a 16-h photoperiod but total daily light input was lower than at NaPPI in spring, illustrate that facility-level differences in light regime and photoperiod are a plausible, though unconfirmed, contributor to the cross-facility reversal of the BABA and KPhi enzyme assignments. With data from two experiments, we cannot attribute the facility-dependent enzyme profiles to a specific environmental variable. No facility-invariant IR treatment- or transgene-specific enzyme signatures could be identified in the comparative analyses across the two facilities.

The observed patterns are consistent with the growth-defence trade-off framework, where allocation trade-offs arise through pleiotropic gene effects and salicylate-jasmonate hormonal cross-talk [2], [8], [9]. BABA imposed the most pronounced morphological and physiological changes; KPhi and the single Rpi line R2A showed minimal morphological changes, whereas blb1 showed a modest height reduction; among Rpi lines, stacked lines showed more pronounced canopy-level dynamics at NaPPI, though the heterogeneity of patterns across individual lines limits the strength of this contrast. By contrast, the brief and intermittent greenness differences, together with the absence of significant Rfd_Lss alterations, across the Rpi lines indicate that stacked and single Rpi gene expression generally does not impose the same sustained photosynthetic or pigmentation-level changes as repeated chemical priming, suggesting that the phenotypic signatures of Rpi gene expression are primarily morphological and metabolic rather than photosynthetic under these conditions. The exception was the coi1 lines, whose higher greenness index, sustained in coi1H1 at PhenoLab and appearing late in the experiment in both lines at NaPPI, indicates that COI1 suppression can additionally alter canopy pigmentation (Supplementary Table S4). These macroscopic patterns were consistent across facilities; the enzyme profiles, however, were not.

In this study, we demonstrated that a phenomics framework integrating chemical resistance inducers, a transgenic collection targeting distinct immunity pathways, and multi-sensor time-series imaging can resolve not only the nature and extent of defence-associated allocation responses, but also which endpoint metabolic signatures are associated with each response. This represents a level of mechanistic resolution not, to our knowledge, previously demonstrated for resistance inducers in a crop system. Three conceptual advances emerge from this work. First, BABA and KPhi induce resistance with mechanistically distinct endpoint metabolic signatures, consistent with distinct reallocation routes. KPhi consistently imposes minimal growth penalties, regardless of facility. The specific endpoint enzyme signature observed is shaped jointly by the resistance mechanism and the growing environment, whereas KPhi's lower cost relative to BABA is conserved across environments. Second, the StCOI1-RNAi lines reveal that JA-regulated carbon allocation is environmentally contingent at the enzyme level even when macroscopic phenotypes are reproducible, evidence for metabolic degeneracy of defence allocation that has implications for multi-environment crop phenomics studies. Third, enzyme-level biosignatures are sensitive to local growing conditions and are not directly comparable across contrasting environments; cross-facility enzyme phenotyping therefore requires explicit characterisation of the metabolic baseline state of control plants. Ultimately, comparing two confounded growing environments helped distinguish which trade-off signatures recurred in both (plant height, KPhi's minimal cost) from those that did not (specific enzyme identities), a distinction that is itself informative for interpreting single-environment phenomics studies.

At the concentrations tested in our study, KPhi showed no detectable growth penalties, while BABA imposed measurable morphological and physiological changes. High-throughput phenotyping platforms thus represent a powerful tool for quantifying potential allocation responses associated with novel resistance genes, resistance mechanisms, or induced resistance strategies, including biological control agents, by enabling precise, non-destructive assessment of plant growth and developmental trade-offs across different experimental systems. However, whether these subtle changes translate to impacts on crop production and yield at larger scales remains to be determined and will require further testing under field conditions across diverse environments and multiple years. Furthermore, field environments simultaneously expose plants to multiple biotic and abiotic stresses, and whether the allocation patterns documented here under pathogen-free, controlled conditions would be expressed, amplified, or overridden under such multi-stress conditions cannot be determined from this study.

### 4.1 Limitations

This study's cross-facility comparison should be interpreted as two independent experiments, not as a replicated test of platform reproducibility. The two experiments were conducted in different growing seasons and under site-specific cultivation regimes. NaPPI and PhenoLab differed simultaneously in pot volume (5 L vs. 2.2 L), growing substrate, photoperiod (18 h vs. 16 h), season (spring vs. autumn), and the associated temperature, humidity, and light conditions reported above. None of these factors were experimentally crossed. Because they covary entirely with facility identity, no cross-facility difference or reversal in this study, including the BABA/KPhi enzyme-association reversal discussed above, can be attributed to a single environmental driver. “Facility” should therefore be read throughout as a proxy for this entire set of confounded factors. With one experiment per facility, we had no statistical basis to isolate a true facility effect from season, substrate, or pot size. A joint Facility × Treatment interaction model could not be fitted, because the block structure differed between facilities. Pot-volume restriction is also known to alter root growth and carbon partitioning, so it remains a plausible, though unconfirmed, contributor to the enzyme-profile differences between facilities. Tubers were assessed at an early stage of tuber filling, about 38 days after transplanting, when the plants were about 7.5 to 8.5 weeks old counting the two weeks of in vitro cultivation. The absence of a detectable cost to tuber number or weight at this timepoint is therefore not evidence that no such cost exists at later bulking stages. In addition, PhenoLab's smaller pot volume likely left tuber development incomplete at this stage. NaPPI's tuber measurements should likewise be read as indicative of yield potential rather than as a fully conclusive assessment.

## Author contributions

**S.P.:** Investigation, Formal analysis, Data curation, Visualization, Writing – original draft. **M.S.:** Methodology, Investigation, Formal analysis, Visualization, Writing – original draft. **P.C.:** Investigation. **J.C.W.:** Data curation, Software, Writing – review & editing. **M.E.M.F.:** Investigation, Formal analysis. **K.Mo.:** Investigation, Software, Data Curation. **E.An:** Conceptualization, Methodology. **L.G.B.:** Conceptualization, Funding acquisition, Supervision, Writing – review & editing. **Å.L.:** Conceptualization, Funding acquisition, Supervision, Writing – review & editing. **T.G.R.:** Conceptualization, Methodology. **K.H.:** Conceptualization, Resources, Writing – review & editing. **E.Al.:** Conceptualization, Supervision, Resources, Funding acquisition, Writing – review & editing.

## Data availability

The datasets generated and analysed during this study are available on Zenodo at https://doi.org/10.5281/zenodo.20671793. Time-series phenotyping data have been pre-processed for outlier removal prior to deposit. The analysis code used for outlier detection, GAMM model fitting, and the enzyme PCA, together with its raw input data and the fitted models, is available in the same Zenodo record (AnalysisCode.zip).

## Declaration of generative AI and AI-assisted technologies in the manuscript preparation process

During the preparation of this work, the author(s) used Claude (Anthropic) to assist in proofreading and editing the manuscript text and the response to reviewers. The analysis code was originally written by the authors; Claude was used to help adapt and extend these scripts for the Zenodo release. The author(s) ran and verified the code and its outputs, reviewed and edited all AI-assisted content as needed, and take full responsibility for the content of the published article.

## Funding

This project was financed by the European Union Horizon 2020 (H2020) project EPPN2020 (grant no. 731013 and TNA #ResPot), the 10.13039/100010665EU H2020 Marie Skłodowska-Curie Actions MSCA-ITN-2017 PROTECTA (grant no. 766048), the Swedish Research Council FORMAS (grant no. 2019–00527), and NordForsk network project NordPlant (grant no. 84597) and project Upscale (grant no. 136986).

## Declaration of competing interest

The authors declare that they have no known competing financial interests or personal relationships that could have appeared to influence the work reported in this paper.
