# Supplementary material for: Multi-facility, high-temporal-resolution phenomics of growth–defence allocation responses in potato
Source: Plant Phenomics. 2026 Oct 22;8(4):100297. doi: 10.1016/j.plaphe.2026.100297 (PMC13744383; doi:10.1016/j.plaphe.2026.100297)
Supplement: Supplementary file 1 [file mmc10.pdf]

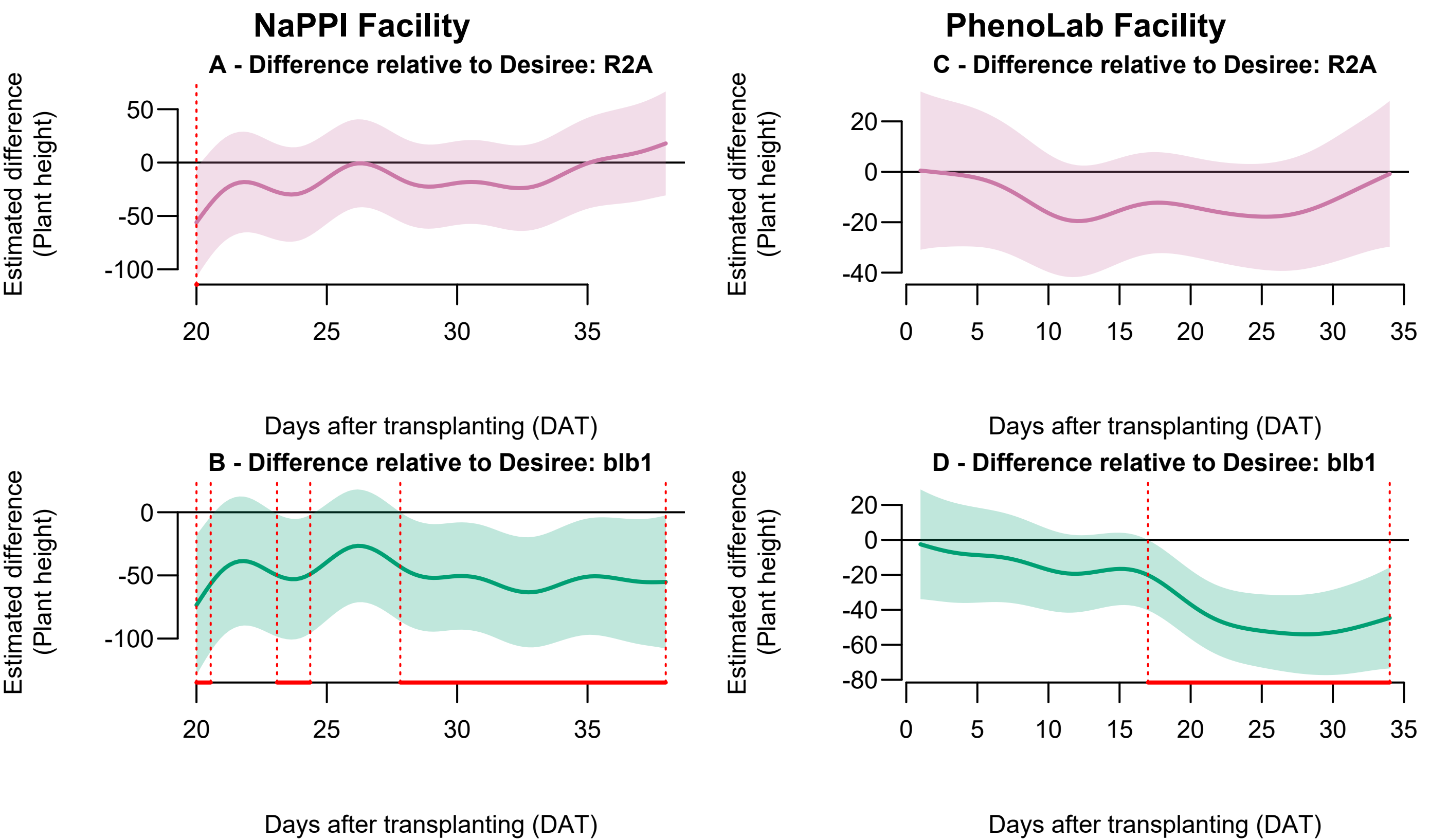

NaPPI Facility
PhenoLab Facility
A - Difference relative to Desiree: R2A
C - Difference relative to Desiree: R2A
B - Difference relative to Desiree: blb1
D - Difference relative to Desiree: blb1
Estimated difference
(Plant height)
Days after transplanting (DAT)
50
0
-50
-100
20
-20
-40
-60
-80
5
10
15
25
30
35
