# Supplementary material for: Multi-facility, high-temporal-resolution phenomics of growth–defence allocation responses in potato
Source: Plant Phenomics. 2026 Oct 22;8(4):100297. doi: 10.1016/j.plaphe.2026.100297 (PMC13744383; doi:10.1016/j.plaphe.2026.100297)
Supplement: Supplementary file 3 [file mmc12.pdf]

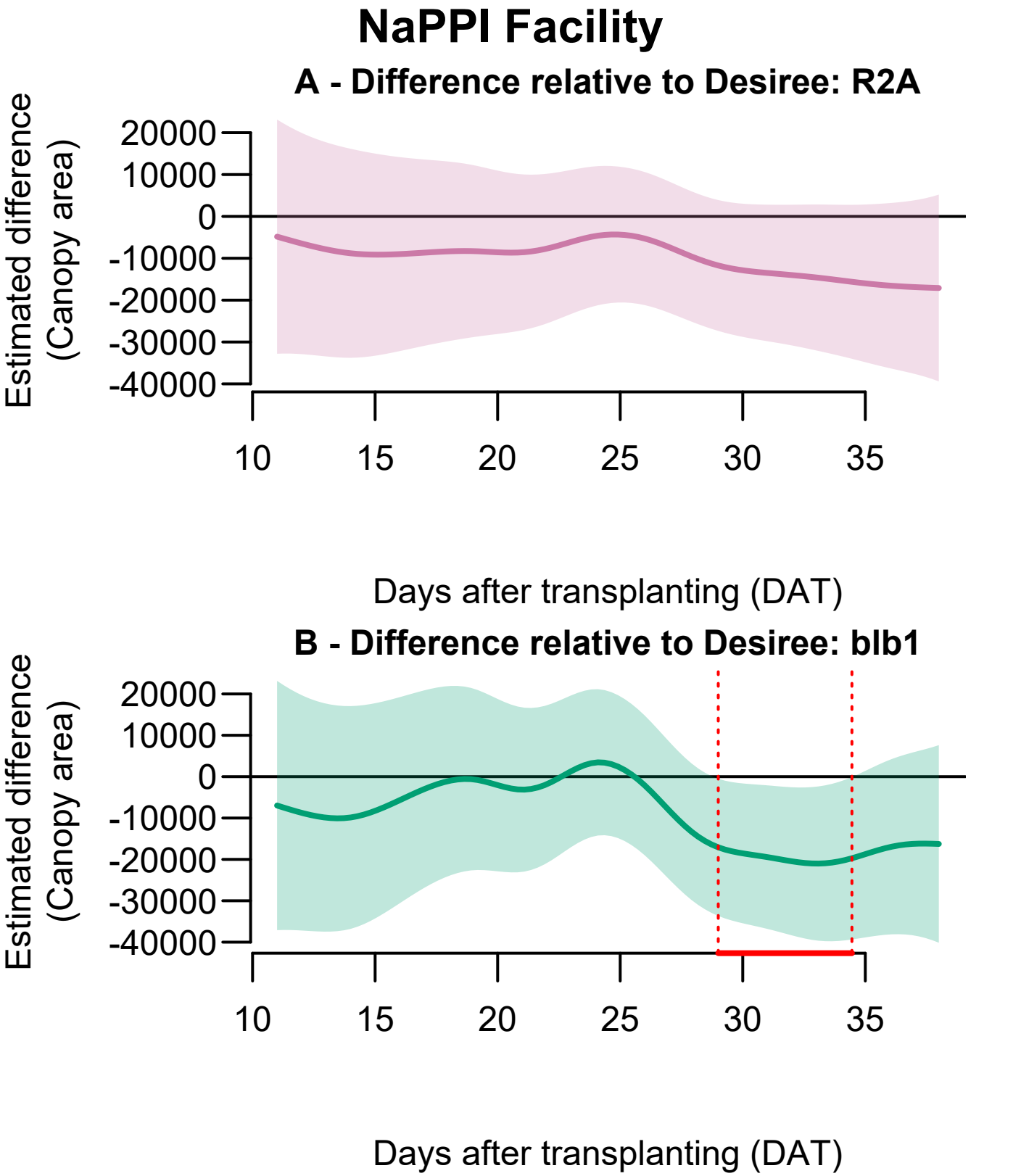
NaPPI Facility
A - Difference relative to Desiree: R2A
Estimated difference
(Canopy area)
20000
10000
0
-10000
-20000
-30000
-40000
10
15
20
25
30
35
Days after transplanting (DAT)
B - Difference relative to Desiree: blb1
Estimated difference
(Canopy area)
20000
10000
0
-10000
-20000
-30000
-40000
10
15
20
25
30
35
Days after transplanting (DAT)
