# Supplementary material for: Multi-facility, high-temporal-resolution phenomics of growth–defence allocation responses in potato
Source: Plant Phenomics. 2026 Oct 22;8(4):100297. doi: 10.1016/j.plaphe.2026.100297 (PMC13744383; doi:10.1016/j.plaphe.2026.100297)
Supplement: Supplementary file 7 [file mmc7.pdf]

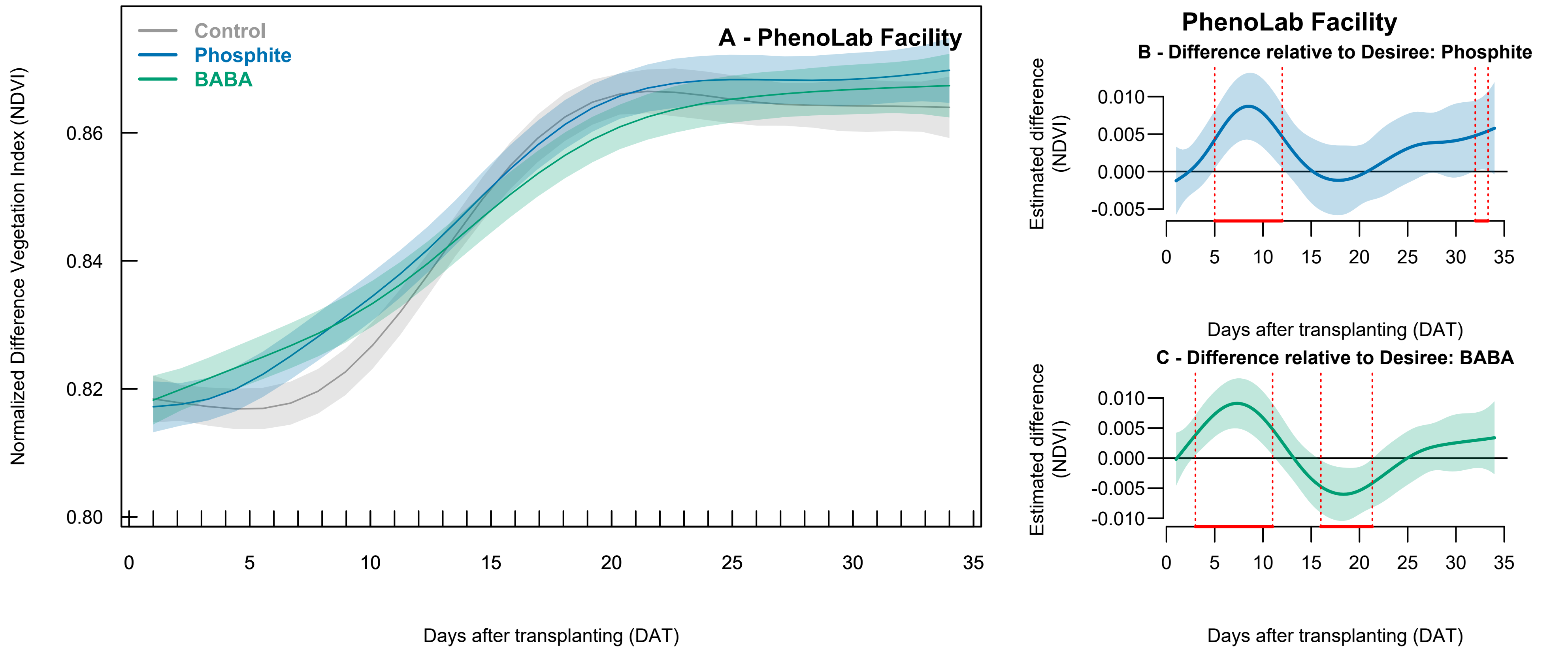
Control
Phosphite
BABA
A - PhenoLab Facility
Normalized Difference Vegetation Index (NDVI)
0.86
0.84
0.82
0.80
0
5
10
15
20
25
30
35
Days after transplanting (DAT)
PhenoLab Facility
B - Difference relative to Desiree: Phosphite
Estimated difference (NDVI)
0.010
0.005
0.000
-0.005
Days after transplanting (DAT)
C - Difference relative to Desiree: BABA
Estimated difference (NDVI)
0.010
0.005
0.000
-0.005
-0.010
Days after transplanting (DAT)
