# Supplementary material for: Multi-facility, high-temporal-resolution phenomics of growth–defence allocation responses in potato
Source: Plant Phenomics. 2026 Oct 22;8(4):100297. doi: 10.1016/j.plaphe.2026.100297 (PMC13744383; doi:10.1016/j.plaphe.2026.100297)
Supplement: Supplementary file 8 [file mmc8.pdf]

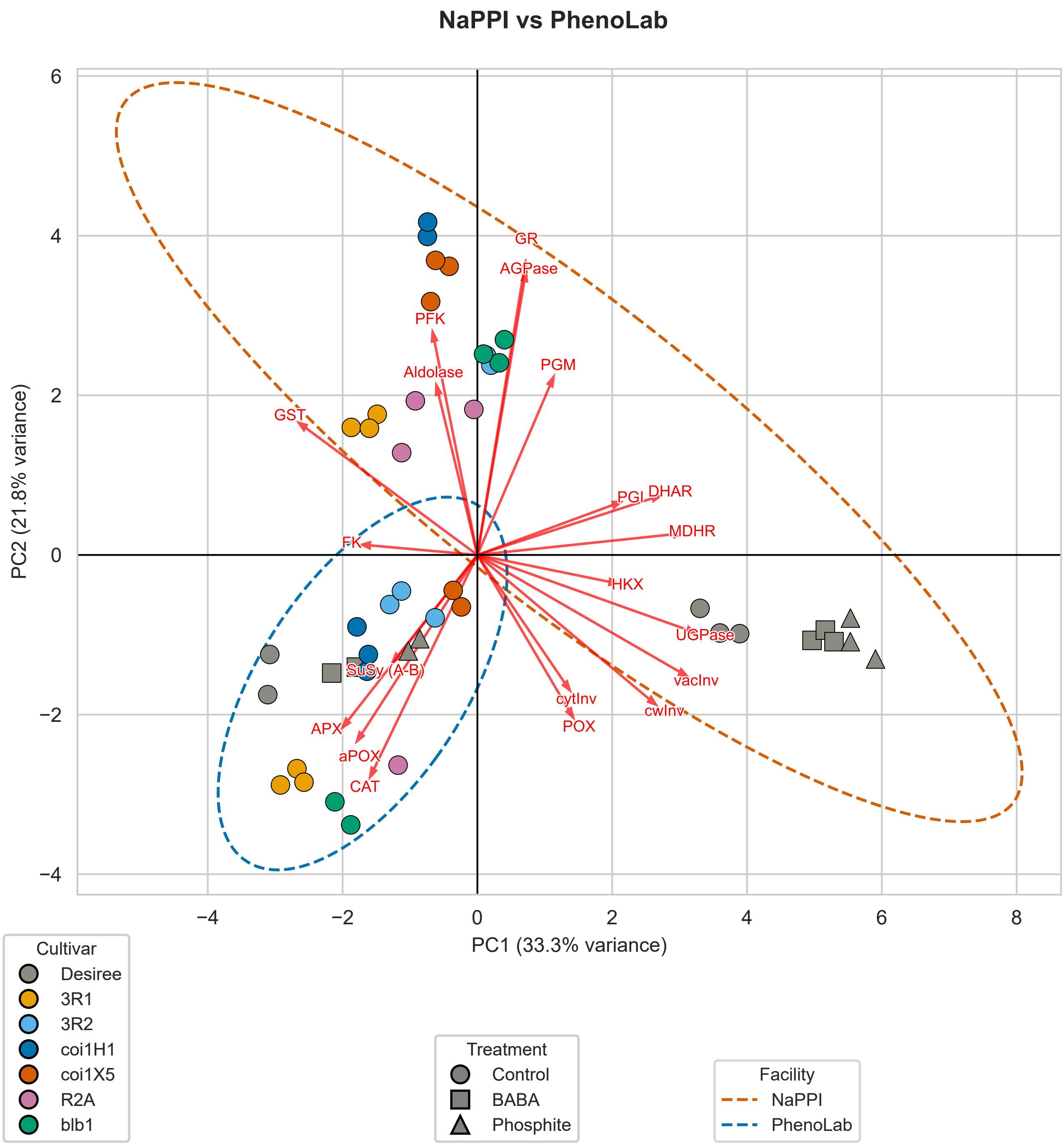
NaPPI vs PhenoLab
PC1 (33.3% variance)
PC2 (21.8% variance)
−4
−2
0
2
4
6
8
GR
AGPase
PFK
PGM
Aldolase
GST
PGI
DHAR
MDHR
FK
HKX
UGPase
SuSy (A-B)
vacInv
cytInv
cwInv
POX
APX
aPOX
CAT
Cultivar
Desiree
3R1
3R2
coi1H1
coi1X5
R2A
blb1
Treatment
Control
BABA
Phosphite
Facility
NaPPI
PhenoLab
