# Supplementary material for: Multi-facility, high-temporal-resolution phenomics of growth–defence allocation responses in potato
Source: Plant Phenomics. 2026 Oct 22;8(4):100297. doi: 10.1016/j.plaphe.2026.100297 (PMC13744383; doi:10.1016/j.plaphe.2026.100297)
Supplement: Supplementary file 9 [file mmc9.pdf]

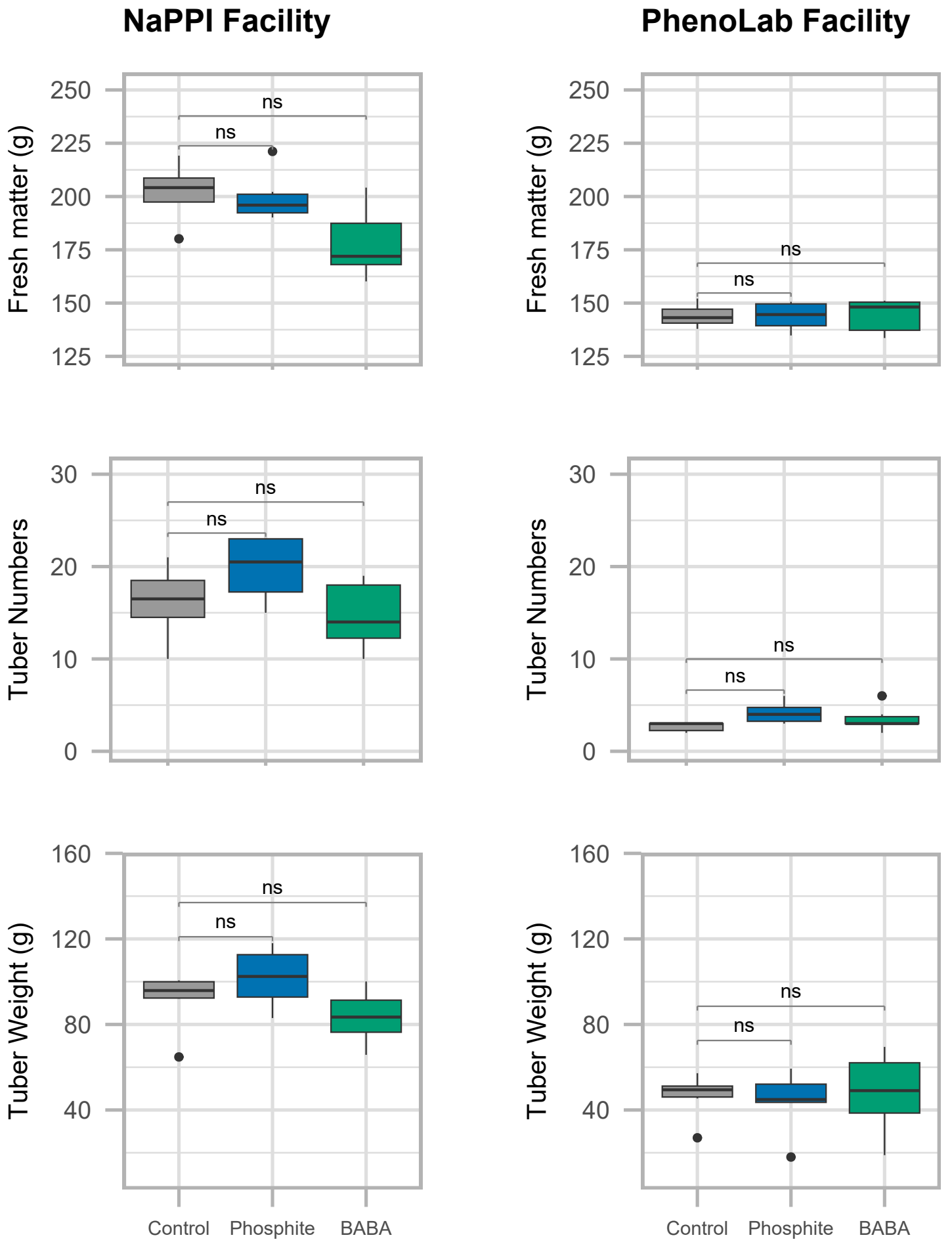

NaPPI Facility
PhenoLab Facility
Fresh matter (g)
Tuber Numbers
Tuber Weight (g)
ns
250
225
200
175
150
125
30
20
10
0
160
120
80
40
Control
Phosphite
BABA
