# Supplementary material for: Multi-facility, high-temporal-resolution phenomics of growth–defence allocation responses in potato
Source: Plant Phenomics. 2026 Oct 22;8(4):100297. doi: 10.1016/j.plaphe.2026.100297 (PMC13744383; doi:10.1016/j.plaphe.2026.100297)
Supplement: Supplementary file 10 [file mmc1.docx]

# Supplemental Table Material

# Supplemental Table S1-S4 Legends

**Table S1.** Endpoint enzymatic activity data used for principal component analysis of induced-resistance treatments and transgenic lines across NaPPI and PhenoLab. The sheet “Individual-enzyme tests” reports one-way ANOVA results for each enzyme within each comparison, with Benjamini-Hochberg-adjusted p-values.

*Table S1 - Endpoint enzymatic activity data used for PCA of induced-resistance treatments and transgenic lines across NaPPI and Phenolab.xlsx*

**Table S2.** Sample-flow and outlier/missing-value summary for every trait with an outlier-detection question: total rows in the published dataset, outlier-detection method and parameters, number of points excluded or missing, and the reason for exclusion. Covers Plant Height, Vegetation Index, and Tuber/Shoot Biomass at both facilities, and Canopy Area and Chlorophyll Fluorescence at NaPPI only.

*Table S2 - Sample-flow and outlier-missing-value summary.xlsx*

**Table S3.** Numerical results for the endpoint comparisons shown in Figs. 7 and 9 and Supplementary Figs. S3 and S9 (dry weight, fresh weight, tuber number and tuber weight): group mean ± SD, effect estimate with 95% confidence interval, and Bonferroni-adjusted p-value for each transgenic line vs. Désirée and each IR treatment vs. Control. The “Method” column gives the model used (linear mixed model with Block as a random intercept, lmer, or linear model, lm) and the “n” column the sample sizes. Means are estimated marginal means for lmer rows and arithmetic means for lm rows, so the difference in means equals the effect estimate.

*Table S3 - Endpoint numerical results for dry weight, fresh weight, tuber number, and tuber weight comparisons.xlsx*

**Table S4.** Sensitivity of the 48 group comparisons shown in Figs. 2-4 and 8 and Supplementary Figs. S1, S4-S6 and S8 to a simultaneous (family-wise) 95% confidence band. Of the 48 comparisons, 42 had at least one pointwise-significant window: 26 remained significant over a narrower window under the simultaneous band and 16 did not. The other 6 had no pointwise-significant window. For each significant comparison, the last column gives the estimated difference and its pointwise 95% confidence interval at the DAT of largest absolute difference, in the units of the trait.

*Table S4 - Simultaneous-CI sensitivity check for GAMM group comparisons.xlsx*
