# Supplementary material for: Multi-facility, high-temporal-resolution phenomics of growth–defence allocation responses in potato
Source: Plant Phenomics. 2026 Oct 22;8(4):100297. doi: 10.1016/j.plaphe.2026.100297 (PMC13744383; doi:10.1016/j.plaphe.2026.100297)
Supplement: Supplementary file 11 [file mmc2.docx]

# Supplemental Figure Legends

**Fig. S1. NDVI dynamics in response to induced-resistance treatments.**

Generalized Additive Mixed Models (GAMMs) were used to analyse NDVI measured in PhenoLab (A). Pointwise differences in the NDVI of phosphite‑treated plants (B), or BABA‑treated plants (C), relative to the Désirée (cv.) control, are shown across days after transplanting (DAT), plotted as treatment minus control (positive values indicate a higher value in the treated plants). Group differences were evaluated using two‑sided pairwise smooth‑difference tests derived from the GAMMs, with significance concluded when the 95% confidence interval of the difference did not include zero. Six biological replicates per treatment were used, and IR treatments were applied weekly via foliar sprays. Control plants are shown in grey, BABA‑treated plants in green, and phosphite‑treated plants in blue. Shaded bands represent the 95% confidence intervals of the GAMM fits. Red lines at the bottom of panels B and C indicate periods during which significant differences at the 95% confidence level were detected.

**Fig. S2. Principal Component Analysis (PCA) of enzymatic profiles combining induced-resistance treatments and transgenic lines across facilities.**

Two-dimensional PCA biplot showing PC1 and PC2 based on endpoint enzymatic profiling from NaPPI and PhenoLab combined into a single analysis. The biplot includes induced-resistance treatments (Control, BABA, phosphite) and transgenic lines (3R1, 3R2, coi1H1, coi1X5, R2A, blb1), together with the control cultivar Désirée. Control plants are shown in grey; transgenic lines are colour-coded as follows: 3R1 (amber), 3R2 (pale blue), coi1H1 (dark blue), coi1X5 (orange), R2A (pink), and blb1 (green) and shaped by treatment (circles: Control; squares: BABA; triangles: phosphite). Dashed ellipses indicate clustering by facility (NaPPI: orange dashed line; PhenoLab: blue dashed line). Red arrows represent loading vectors indicating the contribution and direction of each measured enzyme in the biplot.

**Fig. S3. Yield parameters response to induced-resistance treatments.**

Boxplots showing above‑ground fresh weight (top panels), number of tubers (middle panels), and total tuber weight (lower panels) for plants grown in NaPPI (left panels) and PhenoLab (right panels) at the end of the experiment. Each treatment group includes six biological replicates, except the NaPPI Désirée/Control group’s fresh weight (n = 4; see Supplementary Table S3). Group differences were assessed using two‑sided Dunnett‑type multiple‑comparison tests applied to estimated marginal means (EMMs); none of the comparisons shown reached statistical significance (ns, not significant, throughout).

**Fig. S4. Plant height dynamics in transgenic lines expressing single Rpi genes (R2A, blb1).**

Pointwise differences in plant height of the transgenic lines R2A (pink) and blb1 (green) relative to Désirée (cv.) are shown for NaPPI (A–B) and PhenoLab (C–D) across days after transplanting (DAT), plotted as transgenic line minus Désirée (positive values indicate a higher value in the line). Group differences were evaluated using two‑sided pairwise smooth‑difference tests derived from the generalized additive mixed models (GAMMs), with significance concluded when the 95% confidence interval of the difference did not include zero. Six biological replicates per genotype were used. Red lines at the bottom of each plot indicate periods during which significant differences at the 95% confidence level were detected.

**Fig. S5. Plant height dynamics in PhenoLab for transgenic lines containing stacked Rpi genes (3R1, 3R2) or modified phytohormone pathways (coi1H1, coi1X5).**

Pointwise differences in plant height of the transgenic lines 3R1 (A, amber), 3R2 (B, pale blue), coi1H1 (C, dark blue), and coi1X5 (D, orange) relative to Désirée (cv.) are shown across days after transplanting (DAT), plotted as transgenic line minus Désirée (positive values indicate a higher value in the line). Group differences were evaluated using two‑sided pairwise smooth‑difference tests derived from the generalized additive mixed models (GAMMs), with significance concluded when the 95% confidence interval of the difference did not include zero. Six biological replicates per genotype were used. Red lines at the bottom of the plots indicate periods during which significant differences at the 95% confidence level were detected.

**Fig. S6. Plant canopy area dynamics in NaPPI for transgenic lines expressing single Rpi genes (R2A, blb1).**

Pointwise differences in canopy area of the transgenic lines R2A (A, pink) and blb1 (B, green) relative to Désirée (cv.) are shown across days after transplanting (DAT) in NaPPI, plotted as transgenic line minus Désirée (positive values indicate a higher value in the line). Group differences were evaluated using two‑sided pairwise smooth‑difference tests derived from the generalized additive mixed models (GAMMs), with significance concluded when the 95% confidence interval of the difference did not include zero. Six biological replicates per genotype were used. Red lines at the bottom of the plots indicate periods during which significant differences at the 95% confidence level were detected.

**Fig. S7. Rfd response in NaPPI for transgenic lines containing stacked Rpi genes (3R1, 3R2), modified phytohormone pathways (coi1H1, coi1X5), and single Rpi genes (R2A, blb1).**

Boxplots show the Fluorescence Decline Ratio at light steady state (Rfd_Lss) measured at 34–36 DAT in NaPPI. Six biological replicates per treatment were used. Control plants are shown in grey; transgenic lines are colour-coded as follows: 3R1 (amber), 3R2 (pale blue), coi1H1 (dark blue), coi1X5 (orange), R2A (pink), and blb1 (green). Group differences were assessed using two‑sided Dunnett‑type multiple‑comparison tests applied to estimated marginal means (EMMs); none of the comparisons shown reached statistical significance (ns, not significant, throughout).

**Fig. S8. NDVI dynamics in PhenoLab for transgenic lines containing stacked Rpi genes (3R1, 3R2), modified phytohormone pathways (coi1H1, coi1X5), and single Rpi genes (R2A, blb1).**

Pointwise differences in the NDVI of the transgenic lines 3R1 (A), 3R2 (B), coi1H1 (C), coi1X5 (D), R2A (E), and blb1 (F) relative to Désirée (cv.) are shown across days after transplanting (DAT), plotted as transgenic line minus Désirée (positive values indicate a higher value in the line). Group differences were evaluated using two‑sided pairwise smooth‑difference tests derived from the generalized additive mixed models (GAMMs), with significance concluded when the 95% confidence interval of the difference did not include zero. Six biological replicates per genotype were used. Control plants are shown in grey; transgenic lines are colour-coded as follows: 3R1 (amber), 3R2 (pale blue), coi1H1 (dark blue), coi1X5 (orange), R2A (pink), and blb1 (green). Red lines at the bottom of the panels indicate periods during which significant differences at the 95% confidence level were detected.

**Fig. S9. Yield parameters response in transgenic lines containing stacked Rpi genes (3R1, 3R2), modified phytohormone pathways (coi1H1, coi1X5), and single Rpi genes (R2A, blb1).**

Boxplots showing above‑ground fresh weight (top panels), number of tubers (middle panels), and total tuber weight (lower panels) for plants grown in NaPPI (left panels) and PhenoLab (right panels) at the end of the experiment. Each genotype includes six biological replicates, except: the shared Désirée/Control group’s fresh weight at NaPPI (n = 4); and, for tuber number and tuber weight at PhenoLab, 3R1 (n = 1), coi1H1 (n = 3), blb1 and coi1X5 (n = 4 each), and 3R2 (n = 5) (see Supplementary Table S3). Group differences were assessed using two‑sided Dunnett‑type multiple‑comparison tests applied to estimated marginal means (EMMs). Significant differences between transgenic lines and the control are indicated by asterisks: *p < 0.05, **p < 0.01, ***p < 0.001; ns indicates no significant difference.
